# FURIOUS: Fully unified risk-assessment with interactive operational user system for vessels

**DOI:** 10.1371/journal.pone.0323300

**Published:** 2025-05-28

**Authors:** Yooyeun Kim, Jeehong Kim, Wonhee Lee, Hyunwoo Park, Deuk Jae Cho

**Affiliations:** 1 Graduate School of Data Science, Seoul National University, Seoul, South Korea; 2 Korea Research Institute of Ships and Ocean Engineering, Daejeon, South Korea; Shanghai Jiao Tong University, CHINA

## Abstract

Ship collision risk assessment has advanced over recent years, enhancing maritime safety. However, existing studies often describe ship domains and collision risk assessments in a static manner, lacking interactivity. Interactive visualization of collision risk, especially in multi-ship scenarios has not been sufficiently developed. This gap prompted the development of “FURIOUS: Fully Unified Risk-assessment with Interactive Operational User System for vessels.” This tool aids in visualizing and analyzing collision risk of multi-ship encounter situation through real-time visualization. Our system processes data from Automatic Identification System (AIS). The system performs ship domain calculations and collision risk assessments supported by geographical computations, and includes features like real-time vessel display and collision type detection. Interactive and user-selectable elements, along with dynamic maps enhance real-time decision-making to ensure navigation safety. Additionally, the system aids both experienced and novice users in understanding complicated maritime dynamic environments. Users can adjust parameters like ship type, ship IDs, time window and map type for tailored analyses and proactive collision avoidance. We conducted a user study to validate these features, confirming that they effectively improve situational awareness and enhance decision-making capabilities in real-world scenarios. This paper details the design, implementation, and evaluation of this tool, highlighting its potential to transform maritime decision-making by improving situational awareness and enhancing operational efficiency.

## Introduction

Ship collisions remain a persistent threat, despite ongoing technological advancements aimed at improving safety at sea. The challenge of avoiding collisions is compounded by the complex dynamics of maneuverability of the ship and the nature of marine traffic. Unlike terrestrial vehicles [1], which operate within relatively structured environments, ships must navigate in expansive and unpredictable waters where precise and immediate maneuvering is frequently constrained. Therefore, avoiding ship collisions requires not only the act of evading potential threats but also the capacity to accurately perceive and interpret the surrounding environment.

In the domain of ship collision risk assessment, research has primarily evolved along two main trajectories: collision detection and collision resolution [2]. Collision detection focuses on identifying potential conflicts by predicting ship trajectories and assessing the probability of collision events. This is commonly achieved through motion prediction models and various risk assessment frameworks, such as expert-based heuristics and model-driven probability estimations. On the other hand, collision resolution involves determining appropriate evasive maneuvers, often utilizing optimization techniques, rule-based approaches, or reinforcement learning methods. Many existing systems for autonomous navigation integrate both aspects to provide automated decision-making for Maritime Autonomous Surface Ships (MASS) [3].

Despite these advancements, current approaches exhibit significant limitations. Collision detection methods, while increasingly sophisticated, often rely on assumptions regarding ship behavior and environmental conditions that may not fully capture the complexity of real-world scenarios. Similarly, collision resolution strategies typically focus on prescribing predefined maneuvers, which may not always align with the decision-making preferences of human operators. Consequently, fully autonomous solutions remain challenging, particularly in mixed environments where manned and unmanned vessels coexist [4].

This study takes a different approach. Rather than focusing solely on automating collision resolution, we propose a system that enhances situational awareness for human operators, empowering them to make more informed decisions. By leveraging advanced visualization techniques and real-time data integration, our system provides users with a more intuitive understanding of collision risks and navigational constraints, thus bridging the gap between automated decision support and human judgment. This approach acknowledges the inherent subjectivity in maritime navigation and seeks to assist, rather than replace, the human decision-making process. Our system prioritizes interpretability and user-driven decision-making, ensuring that navigators retain control while benefiting from enhanced risk awareness.

Addressing these gaps, this paper introduces “FURIOUS”, a real-time maritime collision risk assessment system designed to enhance situational awareness for both experienced and novice maritime operators. By providing dynamic and real-time insights into multi-ship encounter, the system reduces cognitive load and the possibility of human error at critical decision-making moments.

“FURIOUS” not only builds on the capabilities of existing visualization tools but also actively engages with the users. The system prioritizes a user-centered design to ensure accessibility for users with varying levels of expertise, and it incorporates a flexible scenario simulation feature that allows users to define and manipulate maritime conditions in real time. This enables a more comprehensive analysis of potential collision scenarios and serves as an invaluable training tool for the navigators. Importantly, the system is designed not to replace human decision-making but to augment it by providing clear, actionable information that supports the expertise of the user.

Our key contributions include:

- The development of “FURIOUS”, a system that enhances situational awareness for marine navigators by providing dynamic, real-time insights into maritime environments, thereby enabling more informed decision-making.
- The introduction of a scenario simulation capability, allowing users to visualize and interact with a wide range of maritime conditions, which is critical for understanding and preparing for potential collision scenarios.
- A user-centered design that ensures the system is accessible and intuitive for users with varying levels of expertise, validated by a comprehensive user study.

## Related work

### Multi-ship collision

Maritime traffic has become increasingly complex with the growing density of vessels in congested waterways and major shipping routes. As a result, the likelihood of multi-ship collision scenarios has risen significantly, making it a critical challenge in maritime safety. Ship collisions not only pose risks to human life but also result in severe environmental and economic consequences, particularly in incidents involving hazardous cargo or high-traffic commercial routes. Given the severe risks posed by ship collisions, many studies have developed and refined strategies for mitigating these risks. This process involves conflict detection, which is guided by the Convention on the International Regulations for Preventing Collisions at Sea (COLREGs) [5], which provides a comprehensive framework of navigational rules to help identify potential collision scenarios. These rules include guidelines for the proper use of navigational lights, signals, and the responsibilities of vessels under various conditions, ensuring that navigators can effectively detect and assess collision risks.

### Challenges in multi-ship encounters

Traditionally, COLREGs were developed based on the assumption of one-on-one encounters between two ships, focusing on collision avoidance in such scenarios. However, applying these principles to multi-ship encounters is challenging, as the rules do not fully account for the complexity of interactions involving multiple vessels [6]. The dynamic nature of multi-ship situations presents significantly more complexity, making it difficult to manage and prevent collisions effectively [7, 8].

### Approaches to multi-ship collision avoidance

To address these challenges, researchers have developed various approaches, which can be broadly categorized into decision-support systems, machine learning-based navigation models, and predictive planning methods.

#### Decision-support systems.

Recognizing the limitations of traditional COLREGs in multi-ship scenarios, Zhang *et al*. [9] proposed a distributed anti-collision decision support system that integrates real-time data and collaborative decision-making among vessels. Their approach enhances situational awareness by allowing ships to exchange information and jointly assess collision risks in complex environments.

#### Machine learning-based navigation models.

Recent advancements in artificial intelligence have introduced adaptive collision avoidance methods based on machine learning. Zhao *et al*. [10] developed a COLREGs-compliant multi-ship collision avoidance system using deep reinforcement learning, which dynamically adapts to changing maritime environments. By learning from various encounter situations, this approach significantly improves safety in real-world applications.

#### Predictive planning methods.

Another line of research focuses on predictive modeling to enhance navigation safety. Zhu *et al*. [11] introduced a prediction-enabled path planning method that leverages real-time data to forecast future vessel positions and adjust courses accordingly. This method reduces collision risks by proactively responding to potential hazards in busy sea lanes. Additionally, Goerlandt *et al*. [12] utilized fuzzy set theory to develop a multi-stage decision-making framework that adapts to varying distances and conditions of multi-ship encounters, offering a robust solution for unpredictable maritime environments.

### Ship collision risk assessment

Assessing the risk of ship collisions involves a systematic approach to identifying potential hazards and quantifying their likelihood and impact [13–15]. Effective risk assessment helps in preventing these accidents by providing navigators and maritime authorities with critical decision-making tools to enhance safety and manage the risks associated with ship operations. The development of ship collision risk assessment has seen a significant transition from traditional methods to more advanced, technology-driven approaches.

#### Traditional geometric approaches.

Initially, the focus was on geometric indicators, such as the Closest Point of Approach (CPA) and Distance to CPA (DCPA), which provided a basic understanding of potential collision risks based on ship positions and velocities. These methods were relatively straightforward, relying on real-time data and manual calculations to estimate the probability of collision based on the trajectory and speed of vessels. However, while useful, these indicators were limited in their ability to account for the complexities of dynamic maritime environments and the human factors involved in navigation.

#### Probabilistic and statistical models.

Over time, these methods have been complemented by more sophisticated approaches that incorporate probabilistic analysis, machine learning, and artificial intelligence. Zhang *et al*. [16] introduced a probabilistic model for collision risk assessment that integrates multiple risk factors, such as ship density, traffic patterns, and environmental conditions. This model enables a more comprehensive analysis of collision risks, considering the variability and uncertainty inherent in maritime operations.

Montewka *et al*. [17] discussed the role of Bayesian networks in modeling the complex interactions between vessels and their environment. These approaches enable a more holistic understanding of collision risks, allowing for better-informed decision-making and more effective risk mitigation strategies.

#### Machine learning-based risk assessment.

Machine learning techniques have also been increasingly applied to ship collision risk assessment. Tritsarolis *et al*. [18] developed a vessel collision risk assessment model using machine learning algorithms to predict collision probabilities based on historical data and real-time inputs. Similarly, Zheng *et al*. [19] utilized Support Vector Machines (SVM) to classify collision risks in congested sea areas, demonstrating the effectiveness of machine learning in enhancing the accuracy of risk predictions.

#### AI-driven intelligent decision support systems.

Artificial intelligence has further advanced the field, particularly through the development of intelligent decision support systems. A framework that combines machine learning with expert knowledge [20] to assess and manage collision risks dynamically was proposed. This system continuously learns from new data, improving its predictive capabilities and providing more accurate risk assessments over time. Additionally, recent studies have explored reinforcement learning-based approaches to enhance risk-aware decision-making in maritime autonomous surface ships (MASS). For instance, Wang *et al*. [21] introduced a safe reinforcement learning framework constrained by COLREGs to optimize collision avoidance decisions, demonstrating improved efficiency and reliability in multi-ship encounter scenarios. Furthermore, Wang *et al*. [22] proposed an anti-collision strategy optimization method using safe reinforcement learning with a hierarchical critic network, designed to enhance the reliability and efficiency of MASS navigation in complex maritime traffic environments.

#### Hybrid models and future directions.

Furthermore, hybrid models that combine traditional and modern techniques are becoming increasingly popular. For example, Goerlandt *et al*. [6] introduced a hybrid risk assessment model that integrates geometric indicators with probabilistic and machine learning approaches. This model provides a multi-layered analysis of collision risks, offering a more robust and adaptable framework for risk management in maritime operations.

The ongoing evolution of ship collision risk assessment reflects the growing complexity of maritime environments and the increasing availability of advanced technologies. As these methods continue to develop, they offer significant potential for improving maritime safety and reducing the incidence of ship collisions. The integration of these diverse approaches into a unified risk assessment framework will be crucial for addressing the challenges of modern maritime navigation.

### Visualization systems for maritime traffic analysis

The field of maritime traffic analysis has seen significant advancements through the development of various visualization systems aimed at enhancing safety and decision-making. However, existing systems often present limitations in terms of real-time interactivity, user accessibility, and scenario simulation flexibility, all of which our system, “FURIOUS", aims to address comprehensively.

For instance, the visual analytic tool developed by Öztürk *et al*. [23] provides a robust framework for analyzing maritime traffic using AIS data, with a focus on both macroscopic and microscopic safety structures. This system is particularly adept at capturing and analyzing historical accident cases, offering a static perspective on collision probabilities. However, in dynamic maritime environments, where multiple vessels interact simultaneously and collision risks evolve in real-time, such a static approach is insufficient. This static approach is one of the fundamental constraints that Zhang *et al*. [24] emphasize in their review of modern maritime safety systems. Our system addresses this limitation by introducing a dynamic, real-time visualization capability that significantly enhances situational awareness for the navigator, enabling more timely and informed decision-making in high-risk scenarios.

In the realm of large-scale data handling, Huang *et al*. [25] presented a GPU-accelerated framework for visualizing extensive vessel trajectories, demonstrating efficiency in managing massive datasets. Although this work represents a powerful approach to data processing, its primary target is users with advanced technical expertise, which may prevent broader adoption. Zhang *et al*. [24] argue that such frameworks must integrate user-centric risk analysis models to be effectively utilized in real-time navigational contexts. Our system, on the other hand, prioritizes user-centered design, ensuring that even those with minimal technical background can navigate and leverage the system effectively. This focus on usability is validated by our comprehensive user study, which highlights our system’s success in bridging the gap between sophisticated technology and practical application, making it accessible to a wider range of users.

Moreover, Feng *et al*. [26] introduced a method for assessing collision risks in real-time, particularly within crowded inland waterways. While their system offers a valuable approach to quantifying collision risks, it lacks the flexibility to simulate various maritime scenarios, a feature that is essential in training and preparedness for potential collisions.

By addressing the gaps in existing systems, “FURIOUS" bridges the gap between the findings from prior studies by incorporating real-time multi-ship interaction modeling, providing navigators with an adaptive visualization environment to support dynamic risk assessment and situation awareness. Unlike traditional collision resolution systems, which prescribe specific avoidance maneuvers, FURIOUS enhances decision-making autonomy, enabling human operators to intuitively interpret and respond to evolving maritime risks.

## Method

### System overview

**Fig 1:**
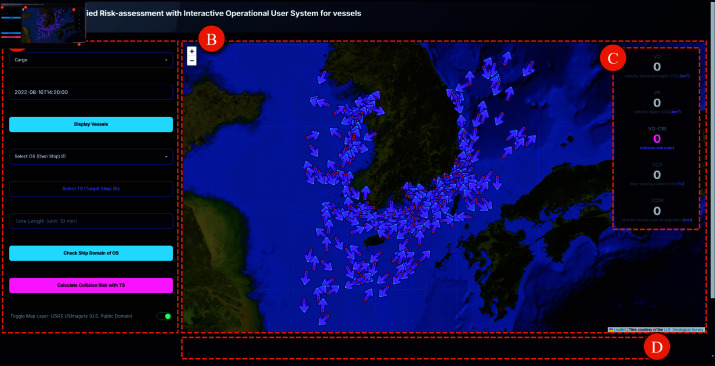
“FURIOUS" system overview. System overview and its main components: (A) Sidebar Control Panel; (B) Main Display Panel; (C) Index Value Panel; (D) Alert Popup Placeholder.

By offering real-time and predictive visualizations, “FURIOUS” is designed to assist the analysis and visualization of marine vessel trajectories and domains in order to assess and mitigate collision risks. The system, depicted in Fig 1, provides visualizations of vessel movements and possible collision scenarios by leveraging data from the Automatic Identification System (AIS).

### Data source

The Automatic Identification System (AIS) is a globally adopted maritime communication system that facilitates the tracking and monitoring of vessels in real-time. AIS data is broadcast by ships and includes essential navigational information such as vessel identity, position, speed, and course. This system plays a crucial role in enhancing maritime safety by enabling the real-time exchange of information among vessels and coastal authorities. The data utilized in this study spans from June 2022 to November 2022, encompassing AIS records from ships in South Korea. The data adheres to the format specified by AIS Hub [27], with additional considerations for Speed Over Ground (SOG) as described by Spire Maritime [28].

In the context of AIS data, several values are critical for understanding vessel movements. SHIP_ID refers to the vessel’s International Maritime Organization (IMO) identification number, ensuring the unique identification of ships globally. RECPTN_DT represents the timestamp of data reception, recorded in Unix time and converted to Coordinated Universal Time (UTC) for human readability. LONGITUDE and LATITUDE are provided in AIS format, where values are recorded in 1/10000 minute increments, necessitating conversion to degrees for standard geographical representation. COG (Course Over Ground) and SOG (Speed Over Ground) are critical navigational parameters, with COG indicating the vessel’s heading relative to true north, and SOG representing the vessel’s speed relative to the Earth’s surface. These values are encoded, where COG and SOG are scaled by a factor of 10, requiring conversion to degrees and knots, respectively, for practical use. Notably, AIS standards stipulate that an SOG of 102.3 signifies unavailable data, a convention adopted to signal technical issues or the lack of transmission.

### Data processing

AIS data is initially ingested by the system and subsequently transformed into GeoJSON format to facilitate geographical operations. The transformation process involves parsing raw AIS data and mapping it to a structured GeoJSON format that includes critical features for maritime analysis. Specifically, each AIS record is converted into a GeoJSON object with properties such as SHIP_ID (unique ship identifier), RECPTN_DT (reception date and time), SOG (speed over ground), COG (course over ground), TYPE (ship type), LEN_PRED (predicted vessel length), and TON (tonnage).

The resulting GeoJSON structure comprises a FeatureCollection containing multiple Feature objects, each representing an individual AIS record. Each Feature object encapsulates both the geometric coordinates, represented as a Point type with coordinates in longitude and latitude, and a set of properties that provide detailed attributes of the vessel.

To ensure the accuracy of length predictions (LEN_PRED), especially in instances where precise tonnage data is unavailable, a linear interpolation method is used. This method leverages static data pertaining to ship type and tonnage to derive a reliable estimate of the vessel length. The transformation process involves the following steps:

- **Data Preparation**: A static dataset containing known ship types, tonnage, and corresponding lengths is loaded. This dataset serves as the reference for interpolation.
- **Length Prediction Algorithm**: For each vessel, the algorithm first filters the static data to match the given ship type, ensuring a case-insensitive comparison. If there is an exact match for the ship’s tonnage within the filtered dataset, the median length of these matching records is returned as the predicted length. If an exact tonnage match is not found, the algorithm performs a linear interpolation by sorting the filtered data by tonnage and calculating the median length for each unique tonnage. The nearest lower and upper bounds around the given tonnage are identified, and the length is interpolated linearly between these bounds by computing the slope of the line connecting them and applying the interpolation formula.
- **Integration into GeoJSON**: Each feature in the GeoJSON data is processed to include the LEN_PRED property. The predicted length is computed using the aforementioned algorithm based on the ship type and tonnage of each feature. The properties within each feature are reordered to insert LEN_PRED in a logical position, ensuring the resulting GeoJSON maintains a structured format. Each feature in the GeoJSON data is processed to include the LEN_PRED property. The predicted length is computed using the aforementioned algorithm based on the ship type and tonnage of each feature. The properties within each feature are reordered to insert LEN_PRED in a logical position, ensuring the resulting GeoJSON maintains a structured format. This comprehensive approach not only standardizes the data for subsequent operations but also enhances the reliability of maritime data analytics. By integrating accurate length predictions into the GeoJSON data, the system supports more precise and effective geographical analysis.

### System architecture

**Fig 2:**
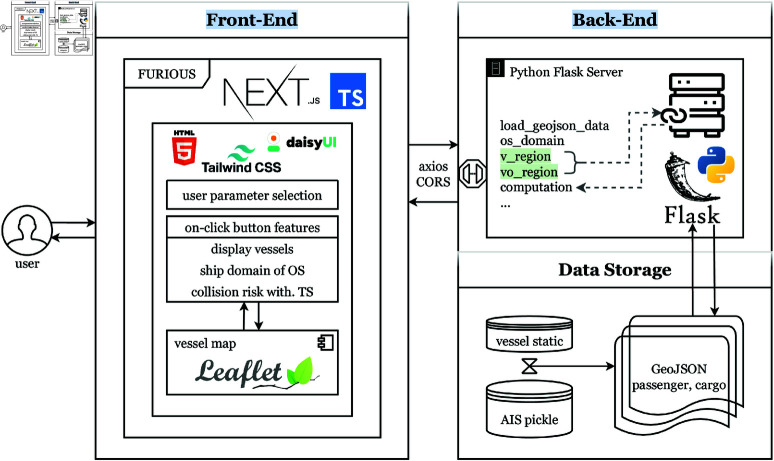
System architecture diagram.

The overall system architecture is illustrated in Fig 2, which provides a visual representation of the different layers and their interactions.

- **Data Storage Layer**: Responsible for data ingestion, preprocessing, and storage. AIS data is transformed into GeoJSON format for efficient spatial querying and visualization.
- **Back-end Layer**: Implemented using Python Flask, which serves data and handles computational tasks. This layer Provides APIs to handle requests from the front-end, performing complex computations such as ship domain calculations and collision risk assessments. Key endpoints of back-end layer include:
  - /load_geojson_data: Loads and filters GeoJSON data based on ship type, date, and time.
  - /get_ship_ids: Retrieves available ship IDs according to user selection.
  - /os_domain: Computes the ship domain areas for the OS.
  - /computation: Calculates collision risk indices and relevant geographical regions.
  - Supporting functions include linear interpolation, GeoJSON data management, encounter mode determination and computation related to ship domain based regions.
- **Front-end Layer**: The front-end is built using Next.js and TypeScript, providing an interactive and responsive user interface for maritime visualization. Tailwind CSS and Daisy UI are employed for user-friendly styling, while React-Leaflet is used for rendering dynamic maps and geographical data. It enables users to interact with the visualization system seamlessly. The front-end layer integrates several key components:
  - Control Panel: This panel allows users to configure and execute specific tasks, such as selecting ship types, dates, and times, and initiating analyses. It serves as the primary interface for scenario configuration and data visualization control.
  - Visualization Module: The module displays real-time vessel positions, ship domains, and collision risk regions. It utilizes React-Leaflet for geospatial visualization, providing a clear and detailed view of maritime scenarios. Additionally, it includes graphical representations of collision risk index calculations, enhancing users’ understanding of potential risks.

### Back-end integration for collision risk assessment

The back-end function for collision risk assessment is based on the methodology proposed by Cheng *et al*. [29]. The following section provides a detailed explanation of the metric used in our system.

### Encounter mode determination

The determination of the encounter mode between two vessels is a critical step in assessing potential collision risks and informing subsequent calculations. This process involves calculating the relative bearing between the Own Ship (OS) and Target Ship (TS) based on their respective courses over ground (COG). The encounter mode is classified into three categories: head-on, crossing, and overtaking, in accordance with the navigation criteria [30]. Specifically, if the relative bearing falls within the range of 112.5° to 247.5°, the scenario is identified as an ‘overtaking’ situation. A relative bearing within 5° to 112.5° or 247.5° to 355° is classified as a ‘crossing’ situation. Otherwise, the encounter is considered ‘head-on’ situation. This classification is essential for accurately determining the ship domain and subsequently computing the collision risk index.

### Ship domain calculation

We adopted a dynamic elliptical ship domain model from Li *et al*. [31] that aligns with COLREGs and typical ship maneuvering behaviors. Unlike traditional models where the ship is positioned at the center of the domain, this model offsets the ship to the lower left of the elliptical domain, reflecting realistic navigation practices.

The size and shape of the elliptical domain vary dynamically based on the ship’s speed and maneuvering characteristics, such as its length. The domain is defined by four parameters: elliptical long semi-axis (*a*), elliptical short semi-axis (*b*), the offset along the major axis (Δa), and the offset along the minor axis (Δb). These parameters ensure that the domain is larger towards the bow and starboard side, aligning with navigators’ collision risk perceptions and COLREGS’ standards.

The elliptical domain is generally aligned with the ship’s velocity vector and incorporates the ship’s speed through a quadrilateral ship domain (QSD) model (Eq 1), which divides the domain into four sectors. These sectors account for the ship’s maneuverability, speed, and course, with the ship’s position anchored at the origin of a Cartesian coordinate system.

$$\text{QSD}=\left\{(x,y)|f(x,y,Q)\leq 1,Q=\left\{R_{\text{fore}},R_{\text{aft}},R_{\text{starb}},R_{\text{port}}\right\}\right\}$$ (1)

Each of the parameters represent the following meaning.

- $R_{\text{fore}}$ / $R_{\text{aft}}$: Longitudinal radii of the QSD in the bow and stern directions of the ship
- $R_{\text{starb}}$ / $R_{\text{port}}$: Transverse radii of the ship

By utilizing the ship’s speed, length, and direction, the calculated parameters effectively capture the dynamics of the vessels. The final elliptic dynamic ship domain model is defined in Eq 2.

$$\frac{(x-\Delta a{)}^{2}}{a^{2}}+\frac{(y-\Delta b{)}^{2}}{b^{2}}=1$$ (2)

$$a=\frac{|R_{\text{fore}}|+|R_{\text{aft}}|}{2},\;b=\frac{|R_{\text{starb}}|+|R_{\text{port}}|}{2}$$ (3)

$$\Delta a=|R_{\text{fore}}|-a,\;\Delta b=|R_{\text{starb}}|-b$$ (4)

**Fig 3:**
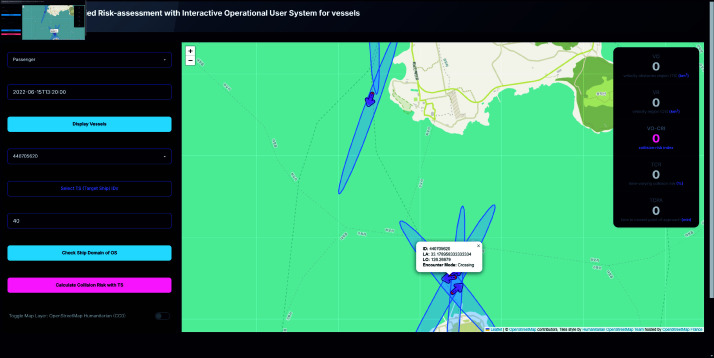
“FURIOUS" system interface. System interface displaying Own Ship (OS)’s elliptical domains of selected time frame.

As illustrated in Fig 3, the system interface dynamically displays multiple elliptical domains of the Own Ship (OS) for each 10-minute interval within the selected time-frame, providing a visual representation of the evolving maritime scenario.

### Region computation considering velocity

To facilitate a comprehensive understanding of the subsequent computational methods, it is crucial to first establish the definitions and formulations of the regions determined by velocity. This foundational knowledge will provide clarity regarding the scope and application of each method described.

**Fig 4:**
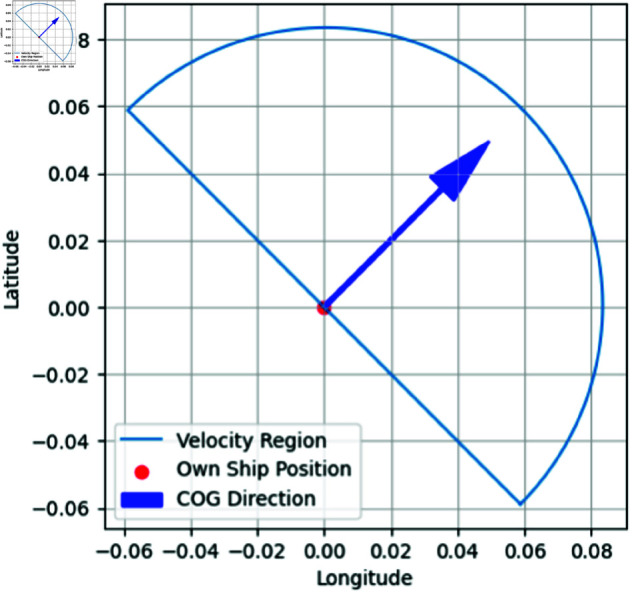
Sample velocity region. Computation implementation of sample velocity region.

- **compute_vo_region**: Velocity obstacle method defines a set of velocities that could lead to a collision if maintained. Its calculation [32] is described in Eq 5 and Eq 6.
  $$VO_{A}|B_{t_{f}}=\overset{\infty }{\underset{t_{f}}{\cup }}\left(\frac{P_{B}(t_{f})}{t_{f}-t_{0}}⊕\frac{ConfP}{t_{f}-t_{0}}\right)$$ (5)
  $$VO_{A}=\overset{n}{\underset{j=1}{\cup }}VO_{A}|B_{t_{f}}$$ (6)
  $VO_{A}|B_{t_{f}}$, calculates the velocity obstacle for object *A* in relation to object *B* at a future time *t*_*f*_. It defines the set of all possible velocities $VO_{A}|B_{t_{f}}$ that could lead to a collision between *A* and *B* if these velocities are maintained.
  The equation involves taking the union (represented by ⋃) of velocity sets over all future times *t*_*f*_. The expression inside the union combines two components:
  - $\frac{P_{B}(t_{f})}{t_{f}-t_{0}}$: This term calculates the relative velocity needed to reach the position of object *B* at time *t*_*f*_ from the current time *t*_0_.
  - $\frac{ConfP}{t_{f}-t_{0}}$: This term represents a confidence region, accounting for uncertainty in the position or movement of *B*.
  The ⊕ symbol denotes the Minkowski sum, which is used to expand the velocity obstacle by adding the uncertainty region. Eq 6 aggregates the velocity obstacles for object *A* across multiple potential encounters with different objects or at different times. The union ${⋃}_{j=1}^{n}$ sums up the individual velocity obstacles $VO_{A}|B_{t_{f}}$ for each possible encounter.
  After defining *ConfP* as a dynamic elliptical model as previously discussed, we utilized the convex hull method to implement the Minkowski sum to merge the regions. The velocity obstacle region is defined as the cumulative area representing the possible positions of target ships. In essence, this region delineates the area that the own ship must avoid entering to prevent potential collisions. Although the original formulation involves a Minkowski addition, what is crucial for our visualization system is information about the boundary of the velocity obstacle region, rather than the detailed coordinates of the entire region. Storing and processing all coordinates corresponding to the entire region would significantly increase computation time and introduce unnecessary complexity. Therefore, when implementing this function in the backend, we employed the convex hull method to efficiently compute and represent the boundary of the velocity obstacle region. The equation for convex hull method is described in Eq 7.
  - **Convex Hull Method**: The convex hull of a set of points represents the smallest convex polygon that can encompass all the points within the set. Mathematically, for a set of points $S=\{p_{1},p_{2},\ldots ,p_{n}\}$ in a plane, the convex hull is expressed as:
    $$\text{Conv}(S)=\left\{\sum_{i=1}^{n}\lambda_{i}p_{i}|\lambda_{i}\geq 0,\sum_{i=1}^{n}\lambda_{i}=1\right\}$$ (7)
    where $\lambda_{i}$ are coefficients representing the convex combination of the points.
- **compute_v_region**: The velocity region is defined as the area within which a vessel can maneuver at a given moment in time. While the exact definition may vary, in our context, it is determined by using the ship’s current speed, which serves as the magnitude of the vector and the ship’s Course Over Ground (COG), which serves as the direction. As shown in Eq 8 [29] where $v_{ship}$ denotes the speed of the ship, we define the velocity region as a semicircle with the radius corresponds to the ship’s current speed. The semicircle is oriented in alignment with the COG of the vessel, with center positioned at the vessel’s current location, as depicted in Fig 4.
  $$V_{region}=\left\{(x,y)|x^{2}+y^{2}\leq v_{ship}^{2}\text{ and }y\geq 0\right\}$$ (8)

### Collision risk index calculation

Time Collision Risk (TCR) represents the proportion of Velocity Obstacle (VO) regions between the own ship and multiple target ships, effectively quantifying the potential collision risk as depicted in Eq 9 [29].

$$TCR(t)=\frac{VO_{\text{region}}(t)}{V_{\text{region}}(t)}$$ (9)

Time to Closest Point of Approach (TCPA) refers to the estimated time before two ships reach their nearest points along their projected paths. Eq 10 [29] considers the distance between the ships, their relative courses, and their relative velocity to determine how long it will take for them to reach the point of minimum separation.

$$TCPA=D_{r}\cos(C_{r}-C_{b}-\pi )/RV$$ (10)

When assessing collision risk based on the variables TCR and TCPA, it is crucial to recognize that their effects differ: while an increase in TCR corresponds to a higher collision risk, a higher TCPA generally implies a lower collision risk. Due to this inverse relationship, rather than using TCPA directly for risk assessment, we modify it using Eq 11 [29] based on the observation period $t_{1}\sim t_{2}$ . The final collision risk index, VO-CRI, is then calculated using Eq 12.

$$TCPA_{i}^{\prime }(t)=\frac{t_{2}-TCPA}{t_{2}-t_{1}}$$ (11)

$$\text{VO-CRI}=TCR\times TCPA_{i}^{\prime }(t)$$ (12)

- **TCR**: Reflects the proportion of Velocity Obstacle (VO) regions between the own ship and multiple target ships, providing a measure of collision risk by aggregating all VO regions from each target ship. It is computed as shown in Eq. 9).
- **TCPA**: Indicates the time remaining until two ships reach their closest points on their respective paths. This variable is fundamental in understanding the temporal aspect of collision risk.
- **Collision Risk Assessment**: While TCR and TCPA are both critical in evaluating collision risk, their influences are opposite; TCR’s increase signals higher risk, whereas TCPA’s increase suggests a lower risk. To account for this, TCPA is transformed as shown in Eq 11 before being integrated into the final collision risk index, VO-CRI, as defined in Eq 12.

### Task analysis

**Fig 5:**
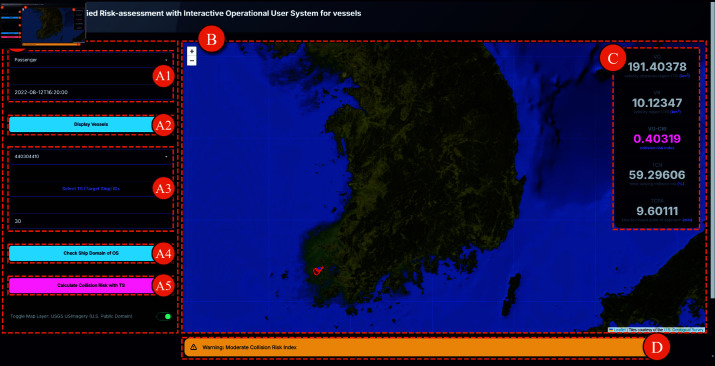
System main interface screen. (A) User Control Panel; (A1) Select Ship Type & Date Button; (A2) Display Vessels; (A3) Select Own Ship & Target Ship; (A4) Check Ship Domain of OS; (A5) Check Collision-Risk Related Index; (B) Main Display; (C) Collision Index Value; (D) Alert Popup.

The system supports three primary user tasks, as depicted in Fig 5. These tasks are accessible via dedicated buttons in the user interface.

### Vessels display

**Fig 6:**
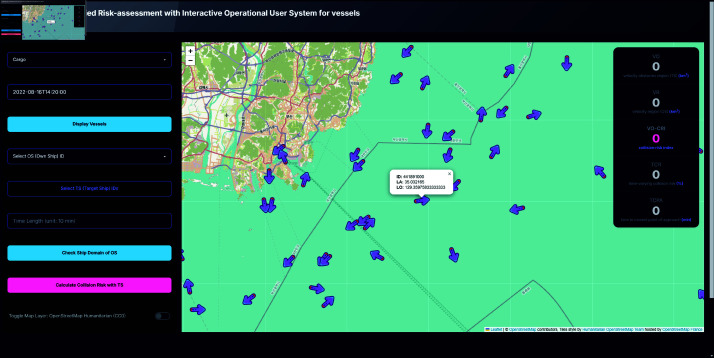
System interface displaying vessels of selected date and time. Toggle of OS vessel shown by clicking the icon.

Users can select a ship type (e.g., cargo, passenger) and specify a date and time to display all vessels of the selected type at the chosen timestamp. Clicking on a vessel marker reveals additional information such as latitude, longitude, and ship ID, facilitating the selection of Own Ship (OS) and Target Ship (TS) IDs, as shown in Fig 6.

### Ship domain of OS

Based on the selected OS ID, one or more TS IDs, and a specified time length, this feature visualizes the ship domain areas for the three encounter scenarios: overtaking, crossing, and head-on, from the selected start time to the end of the specified period. For instance, selecting a start time of 2022-06-13 14:20 and a time length of 90 minutes will show ship domains from 14:20 to 15:50 in 10-minute intervals.

### Collision risk with TS

This feature calculates the collision risk between the OS and multiple TS. The map displays the velocity obstacle regions of the TS and the velocity region of the OS. If these regions intersect, the calculated Collision Risk Index (CRI) is presented as a positive value.

### Visualization and interaction design

The visualization of the “FURIOUS" is designed in alignment with Human-Computer Interaction (HCI) principles to optimize usability, accessibility, and effectiveness [33]. By integrating well-established practices in the field, the system provides an intuitive and interactive experience tailored to the needs of users in the maritime domain. The system’s key features include dynamic maps, interactive elements, contextual information, and alert mechanisms, all of which are strategically incorporated to enhance the decision-making process in real-time maritime navigation.

### Interactive and dynamic mapping

The system leverages React-Leaflet for dynamic geographical data rendering, enabling real-time interaction and potentia4l collision scenarios. The design of the visualization system is guided by the principles of interactive data visualization [34], which emphasize the importance of dynamic querying and immediate feedback, both central to the system’s functionality.

To further enhance user interaction, the system includes a layer toggle feature that allows users to switch between OpenStreetMap (OSM) Humanitarian and USGS Imagery Only layer. The default OSM Humanitarian layer offers a street-map-style interface emphasizing features pertinent to humanitarian efforts, such as water resources, public buildings, and road quality. [35] For more detailed analysis, users can switch to the USGS Imagery Only layer, which provides high-resolution aerial imagery of the United States, with resolutions varying from 6 inches to 1 meter. [36] This feature is particularly useful for users requiring more granular details about the environment, as high-resolution imagery can aid in tasks like navigation and site assessment. The ability to toggle between these layers directly aligns with HCI principles by allowing users to customize their view according to the task at hand, thereby enhancing usability and context awareness [37].

The system also features color-coded markers and buttons, designed to help users intuitively distinguish between Own Ship (OS) and Target Ships (TS). OS-related elements, such as action buttons and regions, are consistently presented in blue tones, while TS-related elements are displayed in red tones. This visual distinction supports cognitive processing by allowing users to quickly differentiate between key entities [38], thus reducing cognitive load and improving decision-making efficiency.

Users can define specific scenarios in multi-ship encounters, offering flexibility for simulating and analyzing diverse marine traffic conditions. Interactive elements, such as tool-tips, popups, and the selector for specific ship IDs, provide contextual information directly within the map interface. For instance, when a user clicks on a vessel marker, detailed information about that vessel, including ID, coordinates, and encounter mode is displayed, offering an overview of the maritime environment. This feature is particularly valuable for academic researchers and ship officers, who require precise and detailed information to assess various maritime scenarios.

### Comprehensibility of advanced metrics

The integration of advanced collision risk metrics, such as the Velocity Obstacle Collision Risk Index (VO-CRI), into user-friendly system is crucial for expanding the usability to a broader audience. VO-CRI is an established metric used for assessing collision risks in dynamic environments, and its effective implementation in user interfaces is essential for enhancing accessibility [29]. The system simplifies the application of these sophisticated metrics by embedding them into an intuitive design, which is chosen to significantly lower the technical threshold required to understand and utilize complex risk assessment tools, making them accessible to users with varying levels of expertise.

The system’s approach aligns with the HCI principles, particularly in reducing cognitive load through well-designed interfaces [39]. By incorporating VO-CRI within a streamlined interface, the system facilitates more efficient and accurate analysis, reducing the time and effort required for users to understand the specific process of calculating collision risks. This is consistent with the broader objective of enhancing user experience by minimizing the complexity of advanced computational tools [40]. The system significantly contributes to making complex metrics understandable and intuitive for users, thus enhancing its utility in real-world applications.

### Intuitive visualization of complex multi-vessel interactions

**Fig 7:**
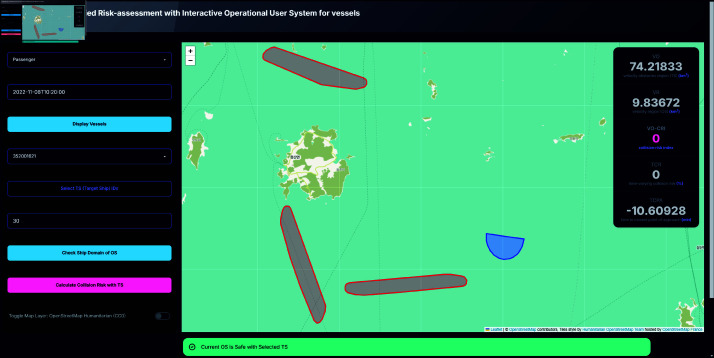
System interface displaying safe scenarios.

**Fig 8:**
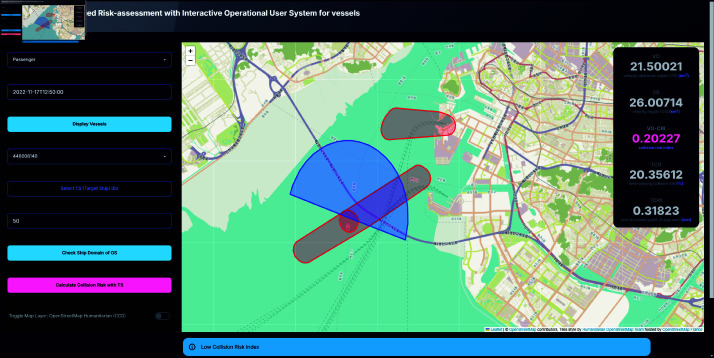
System interface displaying low risk alert scenarios. Alert popup displayed on the bottom of the screen.

A notable advancement of this system is its ability to visualize complex interactions involving multiple vessels in a manner that enhances user comprehension and situational awareness. The system graphically represents ship domains and collision risk areas, through differentiated color-coded regions—red for Velocity Obstacle (VO) regions and blue for Velocity (V) regions. The visual distinction between these regions enables users to easily identify potential collision risks. As illustrated in Figs 7 and 8, the system calculates and displays a Collision Risk Index (CRI) based on the intersecting areas of these regions, with a positive CRI value indicating a potential collision risk.

**Fig 9:**
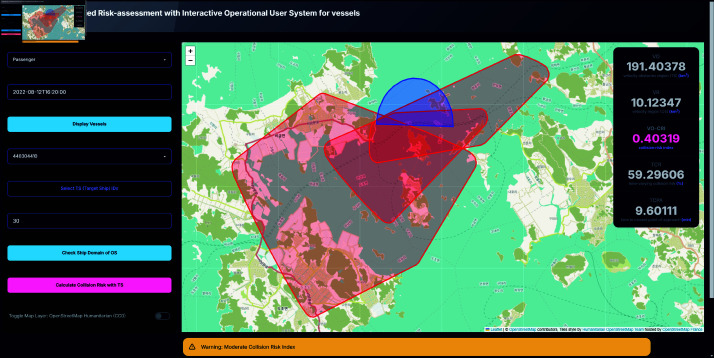
System interface displaying moderate risk alert scenarios. Alert popup displayed on the bottom of the screen.

**Fig 10:**
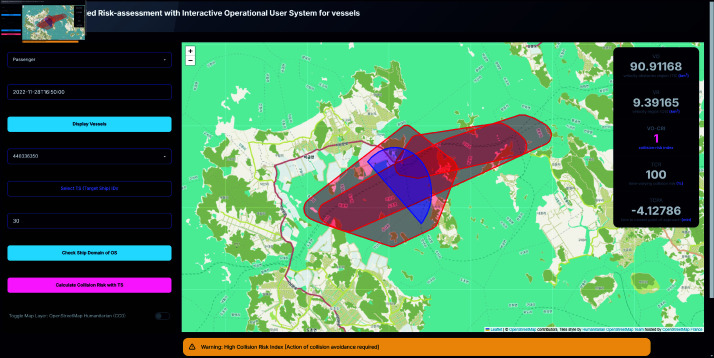
System interface displaying high risk alert scenarios. Alert popup displayed on the bottom of the screen.

To further enhance decision-making of the user, the system includes an alert mechanism that provides real-time warnings based on the calculated CRI. As shown in Fig 9 and 10, these alerts are color-coded according to the severity of the risk:

- High risk condition (0.5≤CRI) triggers a yellow alert, indicating an immediate need for collision avoidance action.
- Moderate risk condition (0.25≤CRI<0.5) also triggers a yellow alert, suggesting caution.
- Low risk condition (0<CRI<0.25) triggers a blue alert, signifying a lower level of concern.
- Safe condition (CRI=0) is indicated by a green alert, confirming that the current OS is safe with respect to the selected TS.

These alert system is designed to be both immediately noticeable and non-intrusive, delivering essential information through clear and prompt visual feedback. This design enables users to efficiently assess and respond to emerging risks, particularly in high-risk or time-sensitive scenarios where rapid and informed decision-making is essential. While the specific thresholds for the CRI used in this system differ, the concept of utilizing a threshold-based approach to risk indication is consistent with the methodology discussed by Hu *et al*. [41] in maritime navigation.

The geographical visualization system exemplifies the successful integration of HCI principles with state-of-the-art maritime risk assessment methodologies. The inclusion of interactive features, such as map layer toggles, scenario settings, and alerts, further enhances system usability by allowing users to customize their view to their specific needs. By presenting complex maritime traffic data in an intuitive interface, the system not only supports comprehensive analysis of maritime traffic but also empowers users to anticipate and mitigate potential collisions, thereby serving as a significant tool in marine trajectory collision analysis.

### Ethics statement

This study involved human participants and was approved by the Institutional Review Board (IRB) of Seoul National University (SNU) [IRB No. 2406/001-009]. The research focused on user interaction for the development of an interactive visualization tool for ship collision regulation. All participants in the user study were properly informed about the purpose and procedures of the research and provided informed consent prior to participation. Participants consented to be involved in the study via written consent forms. All data collected were anonymized to ensure the privacy and confidentiality of the participants.

## Performance analysis

### Empirical validation with historical collision data

To validate the performance of the “FURIOUS" system in assessing collision risks, an analysis using real-world ship accident data was conducted. This evaluation aims to assess whether the system correctly assigns a high value on Collision Risk Index (VO-CRI) to situations that have historically resulted in collisions.

### Dataset and matching approach

The evaluation utilized ship collision accident data obtained from the Maritime Transportation Safety Information System (MTIS) [42]. The dataset was filtered based on the criteria of the original AIS dataset by restricting the records to the period from June to November in 2022 and to vessels classified as cargo or passenger. Due to type compatibility and data availability considerations, the filtered subset comprised of 30 reported collision accidents, of which only 2 were selected for further analysis. The matching process was conducted using accident timestamps and location coordinates, as vessel identifiers were encrypted. The two identified cases involved cargo vessels that experienced collisions. These cases were examined using the system to determine the computed Time Collision Risk (TCR), Time to Closest Point of Approach (TCPA), and Collision Risk Index (CRI) at the moment of collision.

### Experimental results

The system’s collision risk indices for the two verified collision incidents were as shown in Table 1. The results demonstrate that “FURIOUS" assigns significantly high CRI values in real-world collision scenarios. In both cases, the computed index value exceeds the 0.5 threshold which indicates a high probability of collision risk at the respective timestamps, triggering an alert that highlights the immediate need for collision avoidance action. These values confirm that the system effectively captures hazardous navigation conditions.

**Table 1:** Collision risk index analysis for verified cases.

Collision Incident	DateTime	Observation	TCR	TCPA	CRI	Risk Level
“Sanstar Dream” and “Star Ocean”	2022-07-24 15:48	15:30-16:00	50.10519	20.0757	0.50105	High
“Okra 1” and “Kmarin Kenai”	2022-08-20 02:30	02:20-02:40	100	7.58077	1.0000	High

This table provides the computed collision risk indices and metrics used for two verified ship collision cases.

### Statistical analysis

To further validate the system’s effectiveness, we compared the CRI values of the two confirmed collision cases against 100 randomly sampled cases used as a baseline, where actual collision did not occur, from the AIS dataset.

The analysis revealed that the mean CRI for non-collision cases was 0.122, with a standard deviation of 0.068. The 95% confidence interval for non-collision cases ranged from 0.108 to 0.135. In comparison, the Z-scores for the collision cases were significantly higher, with collision case between “Sanstar Dream” and “Star Ocean” having a Z-score of 5.60 and collision case between “Okra 1” and “Kmarin Kenai” showing a Z-score of 12.98. These findings indicate that the CRI values for the collision cases greatly exceed the upper confidence bound (0.145) of non-collision cases, emphasizing the system’s ability to differentiate between safe and hazardous maritime conditions, effectively capturing situations that are prone to collision.

While the results indicate the potential reliability of “FURIOUS" in detecting collision risks, the limited numbers of cases might present challenges in establishing statistical robustness. Given the constraints of available dataset, we acknowledge that the sample size does not allow for extensive statistical validation. Nevertheless, the system’s ability to correctly classify high-risk conditions in both cases suggests that the underlying risk assessment model is aligned with real-world maritime incidents.

### Multi-ship encounter scenario testing

In addition to analyzing individual collision cases, we conducted multi-ship encounter scenario testing to further evaluate the system’s robustness. This testing aimed to validate the system’s capability to handle complex, dynamic interactions involving multiple vessels and provide accurate risk assessments in these scenarios. The rationale for this additional test was to explore whether the system could effectively extend its risk assessment capabilities beyond one-on-one encounters. The testing setup utilized AIS data to simulate these interactions, with the own ship (OS) and multiple target ships (TS) assigned, and risk indices were computed dynamically for each interaction, enabling real-time assessment of collision risks. The results from this multi-ship encounter testing are detailed in the following sections, with additional information and screenshots available at S1 Appendix.

### Two-ship encounter scenario

**Fig 11:**
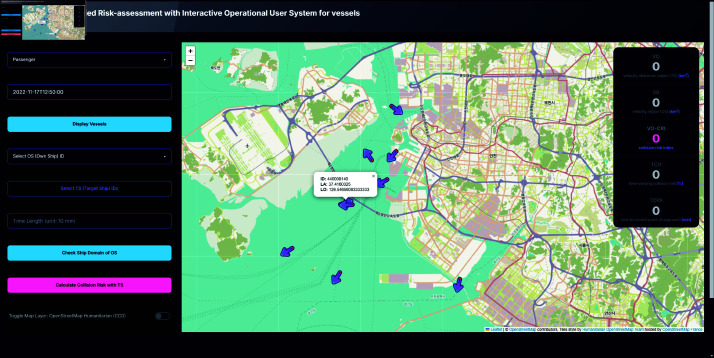
Screenshot of OS icon clicked.

In the two-ship encounter scenarios, two distinct situations were examined. In the first situation, representing a low collision risk, the OS was observed moving upward as it approached the port, while one TS was departing the port in the opposite direction and another TS, although in close proximity to the OS, was moving out of the port, depicted as Fig 11. For this scenario, the system computed a Time Collision Risk (TCR) of 24.82503, a Time to Closest Point of Approach (TCPA) of 0.31823 minutes, and a Velocity Obstacle Collision Risk Index (VO-CRI) of 0.24628. The encounter mode, determined at the time corresponding to the TCPA, was identified as overtaking, which is generally considered less hazardous [43]. This outcome suggests that, despite the close proximity of the vessels, the relative motion and trajectories did not create an immediate high-risk situation.

**Fig 12:**
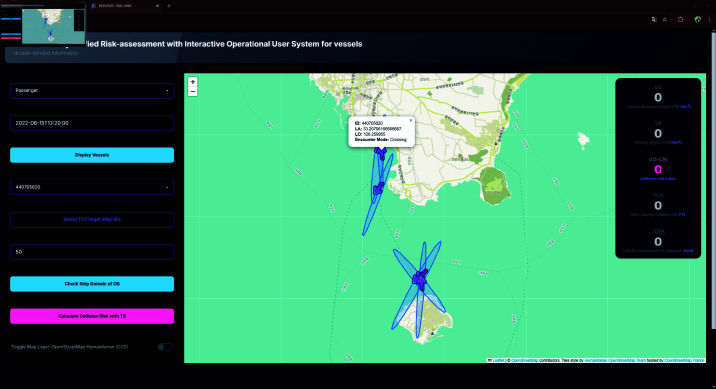
Screenshot of calculated result of encounters at each timepoint.

In a second scenario exhibiting a moderate collision risk, two passenger ships were moving from Gapa Island to Jeju Island, while an additional passenger ship was traveling in the opposite direction from Jeju Island to Gapa Island. In this case, the OS, being one of the vessels on the common route and positioned ahead of the others, resulted in a computed TCR of 29.24976, a TCPA of 6.68918, and a VO-CRI of 0.25337. Although the extended TCPA provided a larger temporal margin before the closest approach, the overlapping trajectories led the system to classify the encounter mode as crossing, depicted as Fig 12. This crossing configuration inherently carries a moderate level of risk, as it reflects the increased potential for conflicting navigational paths. The dynamic computation of these metrics demonstrates that the system can differentiate between various encounter modes and accurately adjust the risk level accordingly.

### Three-ship encounter scenario

The testing was further extended to three-ship encounter scenarios to simulate more complex vessel interactions. In one instance representing a no collision risk situation, the OS was moving toward the left while one TS approached from the right, albeit at a considerable distance and not directly in its path, another TS was moving upward, posing a potential risk only under high-speed conditions, and a third TS was moving away from the OS. Under these conditions, the system registered a TCR of 0, a TCPA of -10.60928 minutes, and a VO-CRI of 0, depicted as Fig 7. The negative TCPA clearly indicates that the vessels had already passed their closest point of approach and were moving apart, thereby confirming the absence of any imminent collision threat.

**Fig 13:**
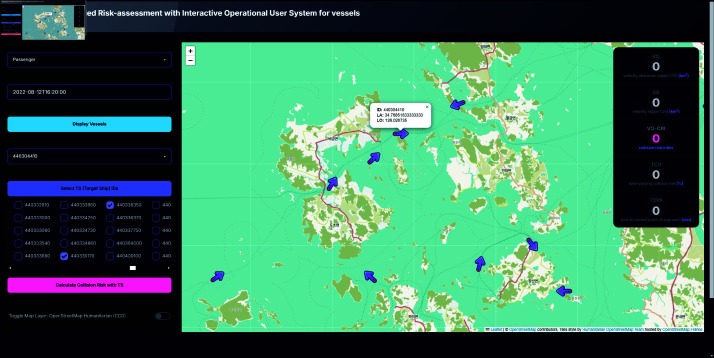
Screenshot of OS icon clicked, while TS being selected.

In a more complex three-ship scenario representing moderate collision risk, the OS was observed heading toward the northeast while one TS approached from the same quadrant and two additional TS vessels followed the OS, depicted as Fig 13. The computed metrics in this scenario were a TCR of 59.29606, a TCPA of 9.60111 minutes, and a VO-CRI of 0.40319. Here, the system detected a combination of crossing and overtaking encounter modes. At the moment corresponding to the TCPA, the OS was expected to be in an overtaking configuration; however, the proximity and coordinated movement of the vessels elevated the overall collision risk. The resulting moderate VO-CRI value underscores the system’s capability to dynamically capture the subtleties of multi-vessel interactions and to signal the need for heightened operator awareness.

**Fig 14:**
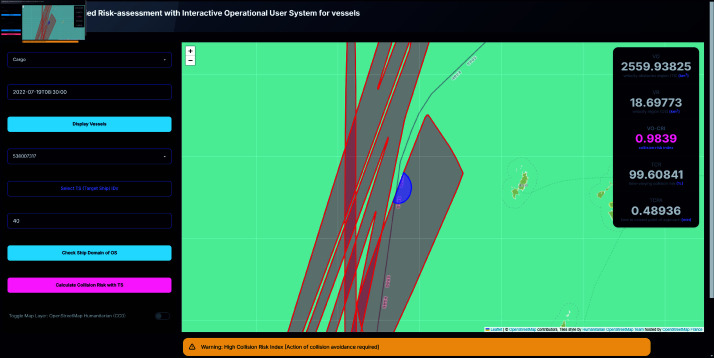
Screenshot of calculated result of collision risk and its metrics.

A high collision risk situation was simulated in a critical three-ship scenario in which the OS was moving in a northwest direction. In this case, one TS was moving in the opposite direction (having already passed the OS), one TS was following the OS in a similar direction, and a third TS was approaching from directly opposite to the OS. The system produced a TCR of 99.60841, a TCPA of 0.48936 minutes, and a VO-CRI of 0.9839, depicted as Fig 14. Although the encounter mode was predominantly classified as overtaking—which is generally associated with lower risk—the particular configuration, especially the TS following the OS whose velocity obstacle intruded into the OS’s safety envelope, resulted in a near-critical risk assessment. This finding demonstrates that even within an ostensibly safe encounter mode, the relative positions and speeds of vessels can culminate in a high collision risk, thereby necessitating immediate corrective action.

### Dense maritime traffic scenario

**Fig 15:**
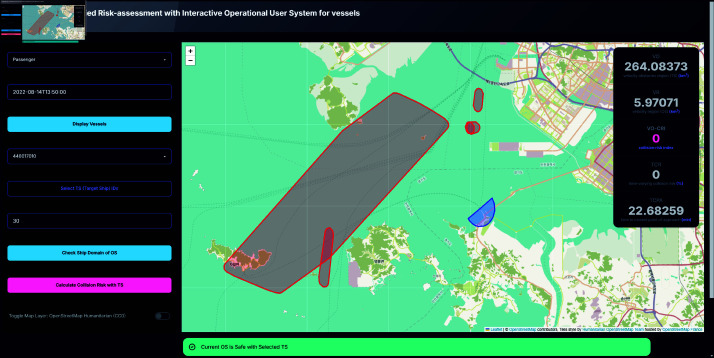
Screenshot of calculated result of collision risk and its metrics.

The final set of tests focused on dense maritime traffic scenarios, simulating conditions typical of port environments. In one scenario considered safe, the OS was navigating into Incheon port while five TS vessels were operating nearby, either maneuvering around the port or exiting it. Under these conditions, the system reported a TCR of 0, a TCPA of 22.68259 minutes, and a VO-CRI of 0, depicted as Fig 15. The extended TCPA and the overtaking encounter mode observed at that moment, along with general reduction of the speed of the ships nearby the port indicated that the vessels maintained sufficient separation, thereby minimizing the risk of collision despite the high density of traffic.

**Fig 16:**
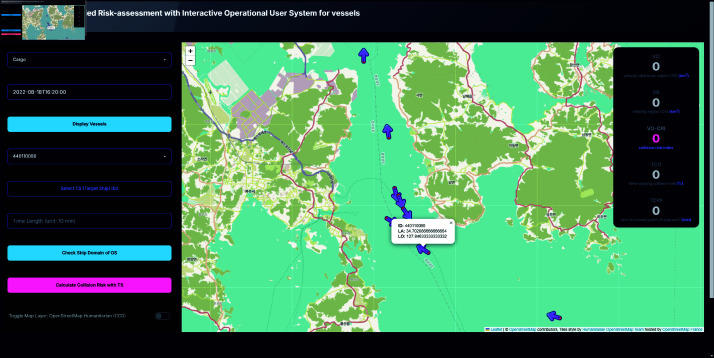
Screenshot of OS icon clicked.

In a contrasting scenario set in a congested port environment at Gwangyang port, the OS was entering the port amid five TS vessels. In this case, one TS was observed entering the port ahead of the OS, another was following the OS into the port, and three TS vessels were departing the port in a direction opposing the OS’s course, depicted as Fig 16. The risk metrics for this scenario were notably higher, with a TCR of 99.99805, a TCPA of 8.1486 minutes, and a VO-CRI of 0.72837. Although the encounter mode was predominantly classified as overtaking, the confined spatial conditions of the port and the converging trajectories of the vessels contributed to a significantly elevated risk index. This result emphasizes that even in encounter modes typically associated with lower risk, environmental constraints and complex vessel interactions can produce conditions that warrant immediate navigational adjustments.

### Results

The results from the multi-ship encounter scenario testing indicate that the “FURIOUS" system is capable of dynamically adjusting risk assessments based on the relative trajectories, speeds, and environmental contexts of multiple vessels. In the two-ship encounters, the system accurately distinguished between low-risk overtaking scenarios and moderate-risk crossing scenarios, as evidenced by the computed TCR, TCPA, and VO-CRI values. In the three-ship encounters, the system was able to capture both non-threatening interactions (with negative TCPA and zero VO-CRI) as well as more complex situations where a combination of encounter modes led to elevated risk indices. Finally, in dense maritime traffic scenarios, the system effectively differentiated between safe conditions—characterized by large TCPA values and zero risk indices—and congested conditions that resulted in high collision risk metrics. Overall, the system’s performance in these varied scenarios suggests that it can provide clear, actionable information to maritime operators, thereby enabling timely interventions such as speed reduction or course alteration to mitigate potential collision hazards. These findings, when combined with the empirical validation from historical collision data, provide compelling evidence of the system’s effectiveness in enhancing situational awareness and supporting safe navigational decision-making in complex maritime environments.

## System evaluation

The primary objective of the user study was to evaluate both the effectiveness and user experience of the “FURIOUS" system. The study sought to gather quantitative and qualitative data on user interactions with the system, focusing on key usability metrics such as ease of use, interface satisfaction, visualization clarity, and overall system responsiveness. A structured survey was administered, incorporating both 5-point Likert scale based close-ended and open-ended questions to capture a comprehensive view of participants. The methodological approach ensured a thorough evaluation of the system’s performance through feedback collected across different user groups was aligned with established.

### User study participants

The study included two main groups of participants to evaluate the system. The first group consisted of five undergraduate students with a foundational understanding of marine environment or geography. This group was selected to represent users with basic knowledge of maritime context, as the system is designed for users with a wide range of expertise. Testing with participants who possess only foundational knowledge ensures that the system remains accessible and functional even for less experienced users. This approach was based on the premise that if users with limited maritime knowledge could effectively navigate and utilize the system, then experts with more advanced understanding would face little difficulties in using it.

The second group comprised ten graduate students with background in visualization, programming, or data science. These participants were chosen for their ability to critically assess the visualization and interactive elements of the system, providing insights into the system’s design and usability from a more technical perspective. The diverse expertise of the participants ensured that the system was evaluated from both the user-friendliness perspective of potential end-users and the technical robustness expected by the experts in visualization and data handling.

### Procedure

### Survey administration

Following a live demonstration of the “FURIOUS" interactive visualization system, user study was conducted via Google Forms. Participants were first introduced to the system’s functionality through the demonstration, after which they completed a survey designed to assess user satisfaction and collect feedback. The survey comprised both closed and open-ended questions to gather comprehensive insights. The survey was conducted from August 8, 2024 to August 12, 2024. Participants provided informed consent through a form embedded in the first section of the Google Forms survey. They had to indicate their consent by selecting the option ‘I agree’ to proceed with the survey. If they chose ‘I do not agree,’ the survey would automatically terminate. The consent obtained was documented electronically.

### Question design

The survey covered several key areas relevant to Human-Computer Interaction (HCI) and User Interface/User Experience (UI/UX) design principles [44][45]. These included:

- Ease of Use and Accessibility: Participants were asked to rate the system’s ease of use and the clarity of instructions provided. This aligns with HCI principles concerning usability and user-friendly interface design, ensuring that the system is intuitive and accessible to users with varying levels of technical expertise.
- Interface Satisfaction: Questions focused on the intuitiveness and clarity of the system’s interface. These questions are grounded in UI/UX principles that emphasize the importance of a clear, logical, and visually appealing interface to enhance user satisfaction and effectiveness.
- Visualization Comprehensibility: Participants evaluated the clarity and usefulness of the visualizations, a critical aspect in HCI for ensuring that complex data is represented in a way that is easily interpretable and actionable.
- System Responsiveness: The survey also included questions about the system’s performance, particularly its responsiveness, which is essential for a smooth user experience, especially in real-time applications like maritime risk assessment.
- Overall Satisfaction and Future Use: Finally, the survey gauged overall satisfaction and willingness to recommend or reuse the system, which are important indicators of user acceptance and the system’s potential impact.

This structured approach ensured that the feedback collected was comprehensive and aligned with established HCI and UI/UX frameworks, providing valuable insights for further refinement of the system. Questions are available at S2 Appendix.

### Results and discussion

**Fig 17:**
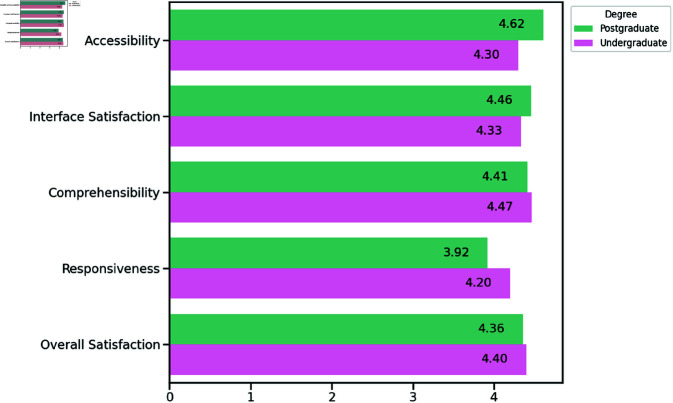
Summary statistics of close-ended questions. Topics of average ratings: (A) Usability and Accessibility; (B) Interface Satisfaction; (C) Comprehensibility; (D) Responsiveness; (E) Overall Satisfaction.

Overall, the results from the user study, depicted as Fig 17, indicate that the system was well-received across both participant groups—undergraduate students with foundational knowledge of marine environments and graduate students with technical expertise in visualization and data science. The diverse backgrounds of the participants provided a comprehensive assessment of the system from both a user-friendly and a technical standpoint.

### Ease of use and accessibility

Usability emerged as a strong point for the “FURIOUS". Postgraduate participants rated this category with a mean score of 4.62 out of 5. The undergraduate group, however, provided a slightly lower but still positive rating of 4.30. This difference highlights that while the system is intuitive for all users, postgraduate participants, likely due to their greater experience with technical systems, found it even more accessible. The clear instructions given in advance of the live demo and ease of interaction were consistently praised across both groups, suggesting that the system’s interface design effectively meets the needs of users with varying levels of technical expertise.

### Interface satisfaction

Interface satisfaction yielded similarly positive results between both groups, with postgraduate users assigning an average score of 4.46, while undergraduates gave it a 4.33. Both groups acknowledged the clarity and simplicity of the system’s interface. Postgraduate participants, with a larger experience in UI/UX design, provided suggestions for improvement, such as optimizing the navigation methods for vessel selection and enhancing the guidance for layout. These suggestions underscore opportunities for refining the interface in future iterations to enhance overall user engagement and satisfaction, particularly for more advanced users.

### Visualization comprehensibility

Another area in which the system performed well was visualization comprehensibility, with undergraduates giving the highest score of 4.47 and postgraduates slightly lower at 4.41. Both groups affirmed that the system’s visual representations were clear and effective. Undergraduates particularly appreciated how complex data of maritime environments was presented in an intuitive and understandable manner including corresponding analysis results, allowing them to grasp key concepts with relative ease. These findings reinforce the system’s success in balancing comprehensive data visualization with simplicity, making it accessible even to users without advanced domain knowledge.

### System responsiveness

System responsiveness received mixed feedback, with postgraduates rating it lower at 3.92, while undergraduates gave it a higher score of 4.20. The lower rating from the postgraduate group likely reflects their heightened expectations for real-time data handling and system efficiency, based on their experience of visualization systems. Although both groups observed slight lags when dealing with large datasets, the undergraduate group did not consider it to be a critical issue. The system’s ability to provide timely information, allowing users to make swift decisions, despite minor delays, was still acknowledged as sufficient for real-time decision-making in maritime contexts, and further optimization of performance could be explored in future updates.

### Satisfaction and future use

Finally, overall satisfaction was consistently high across both groups, with undergraduates assigning a score of 4.40 and postgraduates rating it 4.36. The feedback collected indicated strong potential for future use, with participants expressing a willingness to recommend the system to others. The system’s ease of use, combined with its ability to handle complex data visualizations, positions it as a valuable tool for both novice users and professionals in maritime risk assessment. This broad acceptance highlights the system’s adaptability and its capacity to serve users from various backgrounds.

The feedback gathered from the user study provides clear indications of both the strengths and areas for improvement of the system. The insights from participants will be integral in guiding future developments, particularly in enhancing the interface design and expanding the system’s capabilities. By incorporating this feedback, “FURIOUS" can be further refined to better meet the diverse needs of its users, ultimately contributing to safer and more efficient maritime operations.

## Conclusion

In this study, we introduced “FURIOUS”, a real-time maritime collision risk assessment system that enhances situational awareness by providing dynamic insights in multi-ship encounter situation. The system leverages AIS data and advanced visualization techniques to contribute to safer and more efficient maritime navigation. The system allows for efficient spatial querying and visualization, offering users a clear representation of the maritime environment. The system facilitates real-time analysis of vessel trajectories and provides flexible scenario simulations, making it a valuable tool for understanding and mitigating collision risks. Its user-friendly interface and dynamic visualization ensure accessibility for users with varying levels of expertise, thereby enhancing practical usability in the field. Feedback from the user study we conducted reinforced these strengths, particularly praising the system’s intuitive design and clear visualizations. “FURIOUS" proves to be a valuable tool for understanding and mitigating collision risks in high-density maritime settings.

However, we acknowledged the system has following limitations when being applied to the real-world context. Its reliance on AIS data, while providing real-time tracking, is subject to the quality and availability of the data, which can affect the system’s performance. Additionally, the current system, while robust in assessing collision risks, does not fully address the complexities of human decision-making under pressure or integrate all potential environmental factors, such as sudden weather changes or mechanical failures.

Future work will focus on expanding the system’s capabilities by incorporating additional data sources, such as weather forecasting, to improve the accuracy and reliability of collision risk assessments. Moreover, enhancing the system’s responsiveness and updating the calculation metrics to handle large geographical datasets and reflect the latest advancements in maritime collision risk assessment will be considered. By incorporating more sophisticated algorithms and refining the visualization and user interaction components, “FURIOUS" would be a more versatile decision-supporting tool that is well-equipped to handle the evolving studies of maritime navigation in increasingly dynamic environments. These improvements will ensure that the system remains at the forefront of maritime safety technology, adapting to new challenges as they arise.

## Supporting information

S1 Appendix Screenshots of Multi-ship encounter scenario testing. (PDF)

S2 Appendix User Survey. This appendix contains the translated questions from the user survey conducted as part of the system evaluation of the “FURIOUS".
(PDF)

S3 Appendix Author-Generated Code. This appendix provides a compressed ZIP archive containing the code developed by the authors. The same code is also accessible from the GitHub repository specified in the Code Availability section.
(ZIP)
