# Supplementary material for: FURIOUS: Fully unified risk-assessment with interactive operational user system for vessels
Source: PLoS One. 2025 May 28;20(5):e0323300. doi: 10.1371/journal.pone.0323300 (PMC12118981; doi:10.1371/journal.pone.0323300)

Supporting Information: Appendix

Screenshots of Two-ship Encounter Scenario

Ship Type	OS ID	TS IDs	Observation
passenger	440008140	440001360, 440017940	2022-11-17 12:50 ~ 13:30

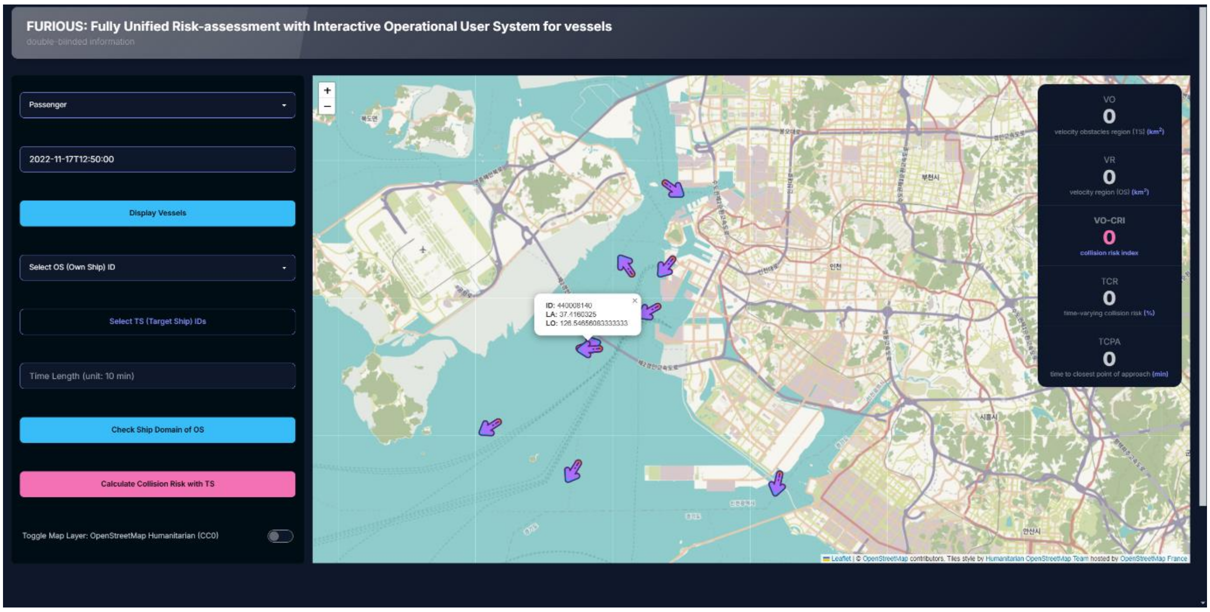

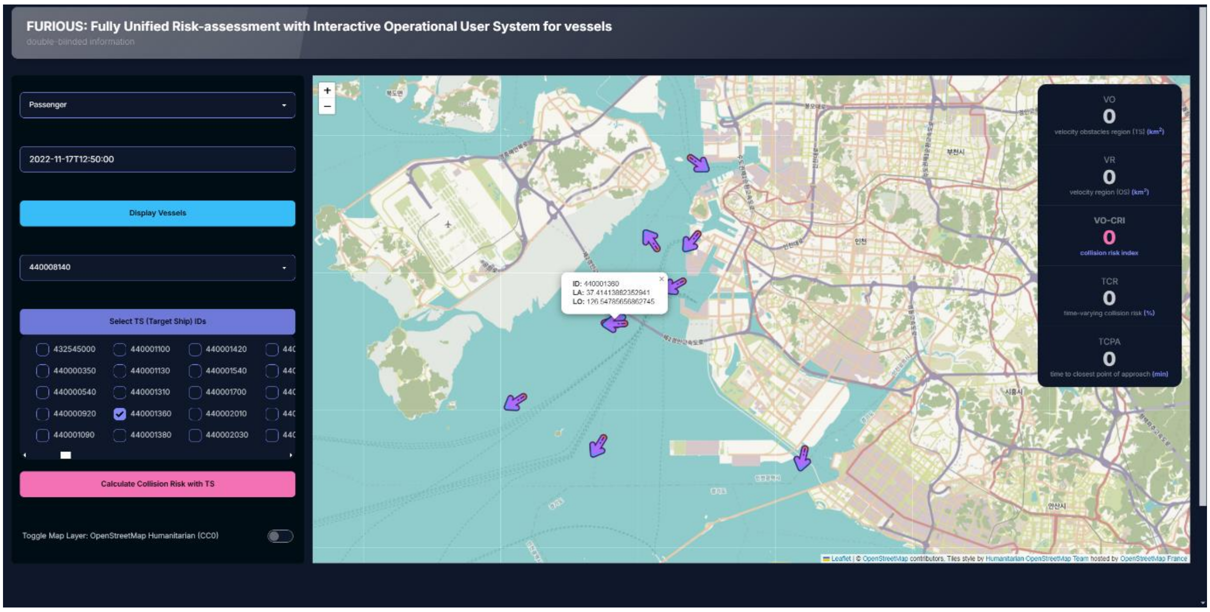

Supporting Information: Appendix

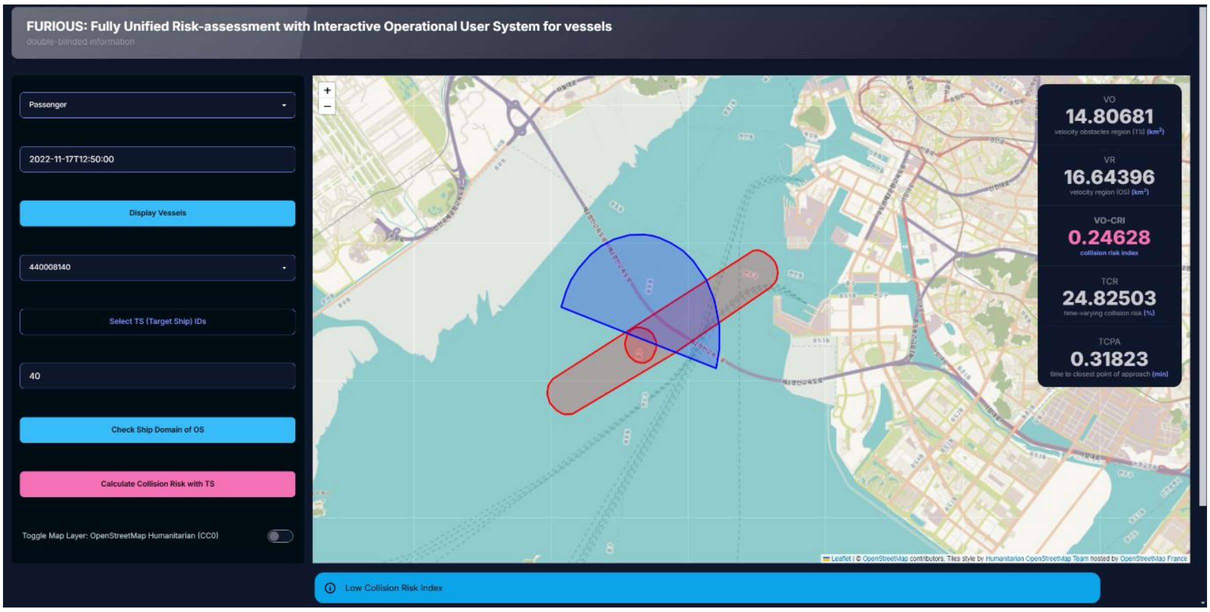

Ship Type	OS ID	TS IDs	Observation
passenger	440705620	440702480, 440705860	2022-06-15 13:20 ~ 14:10

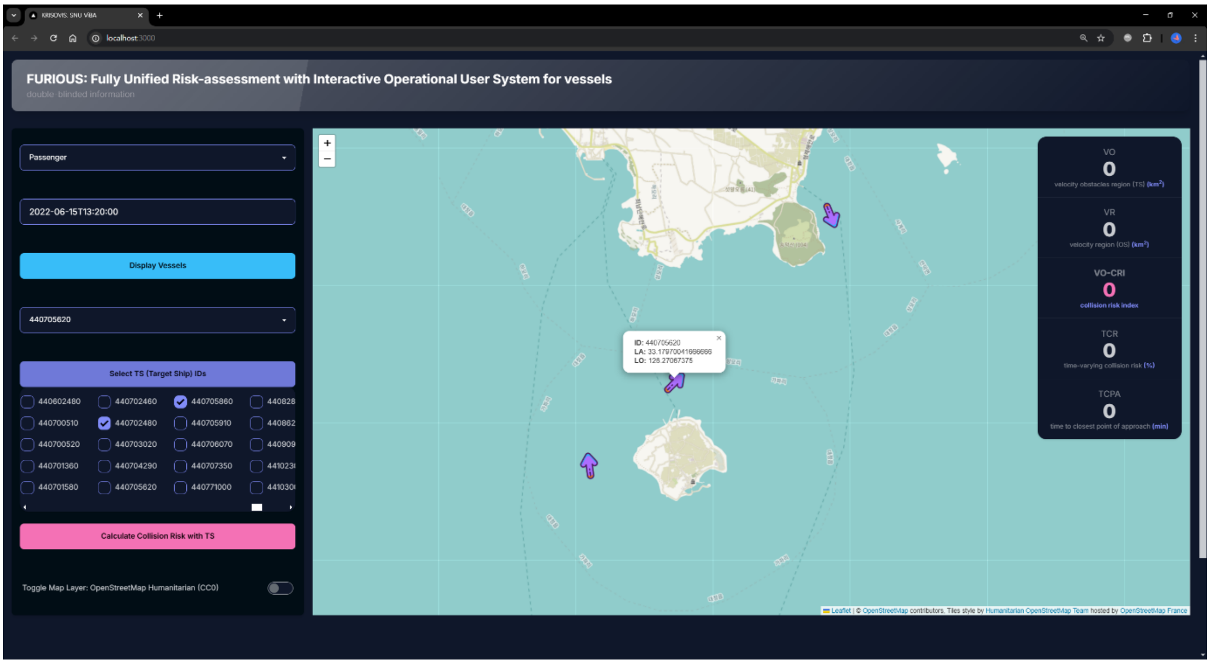

Supporting Information: Appendix

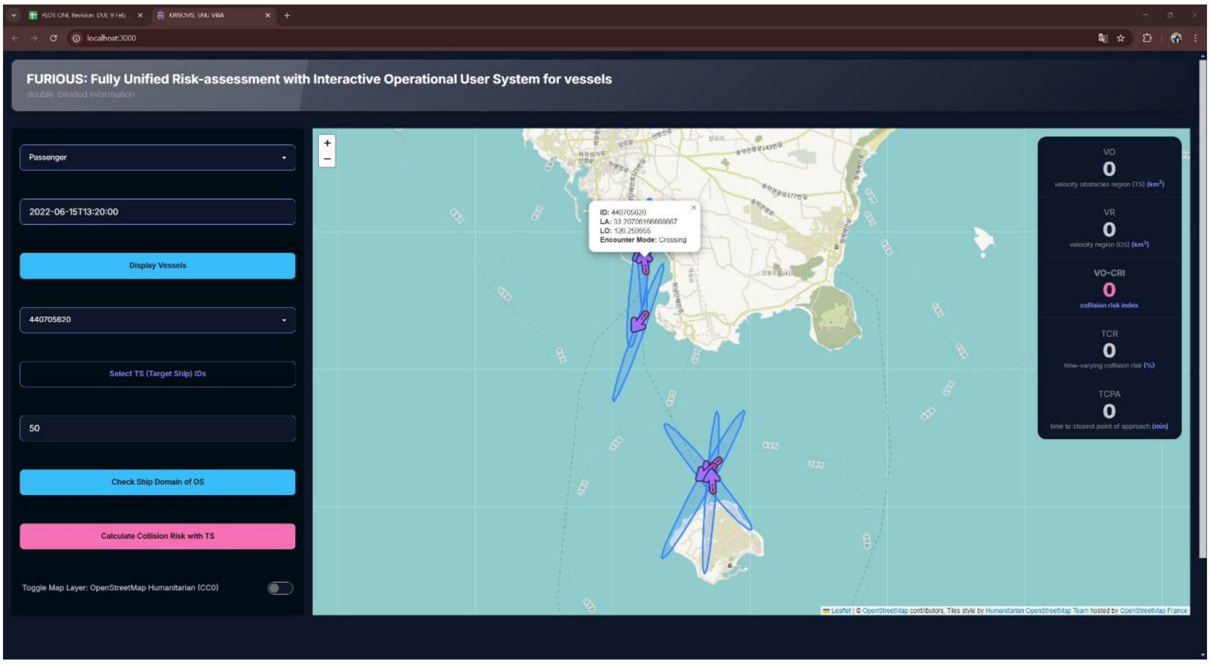

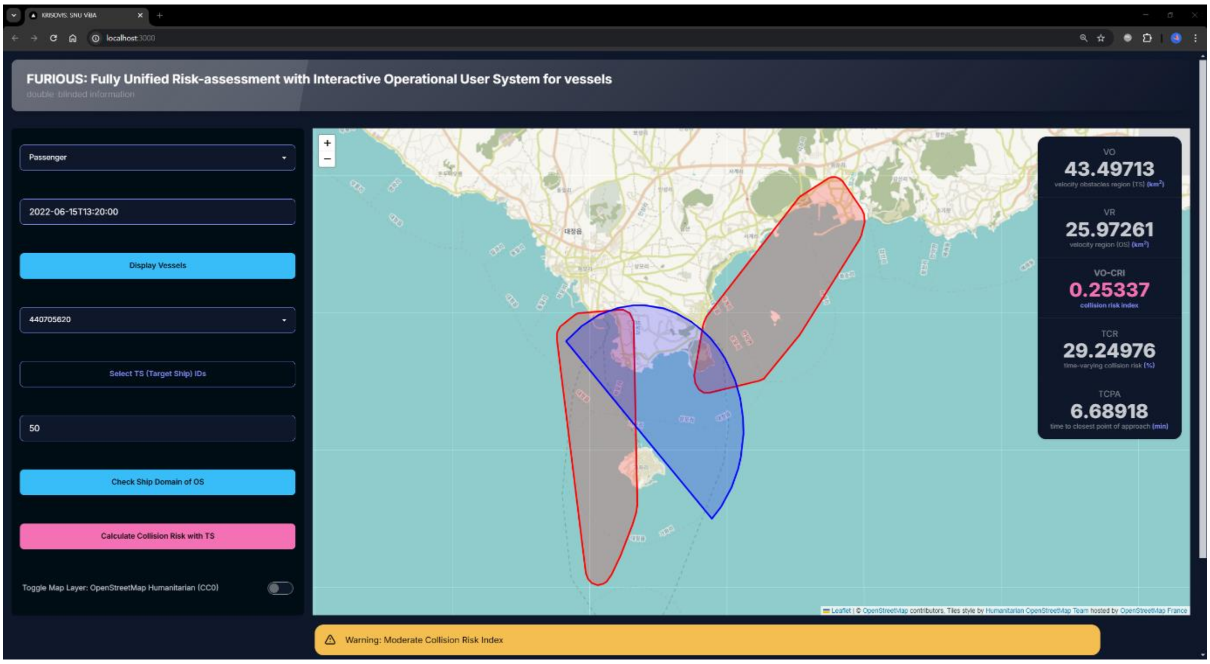

Supporting Information: Appendix

Screenshots of Three-ship Encounter Scenario

Ship Type	OS ID	TS IDs	Observation
passenger	352001621	440232000, 440304270, 440301720	2022-11-08 10:20 ~ 10:50

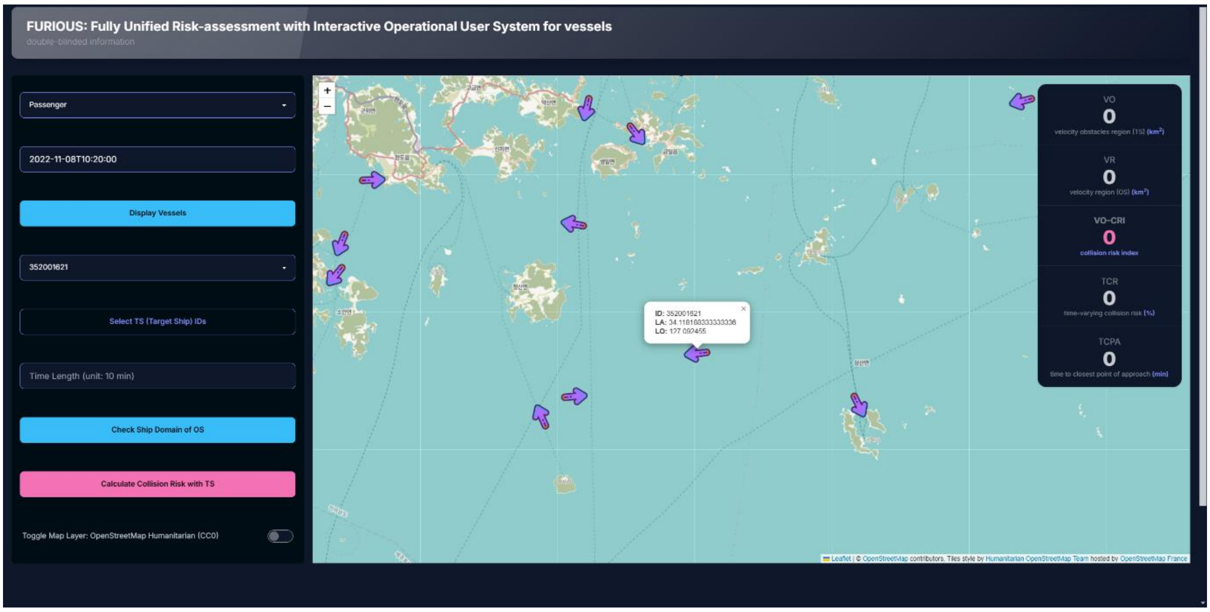

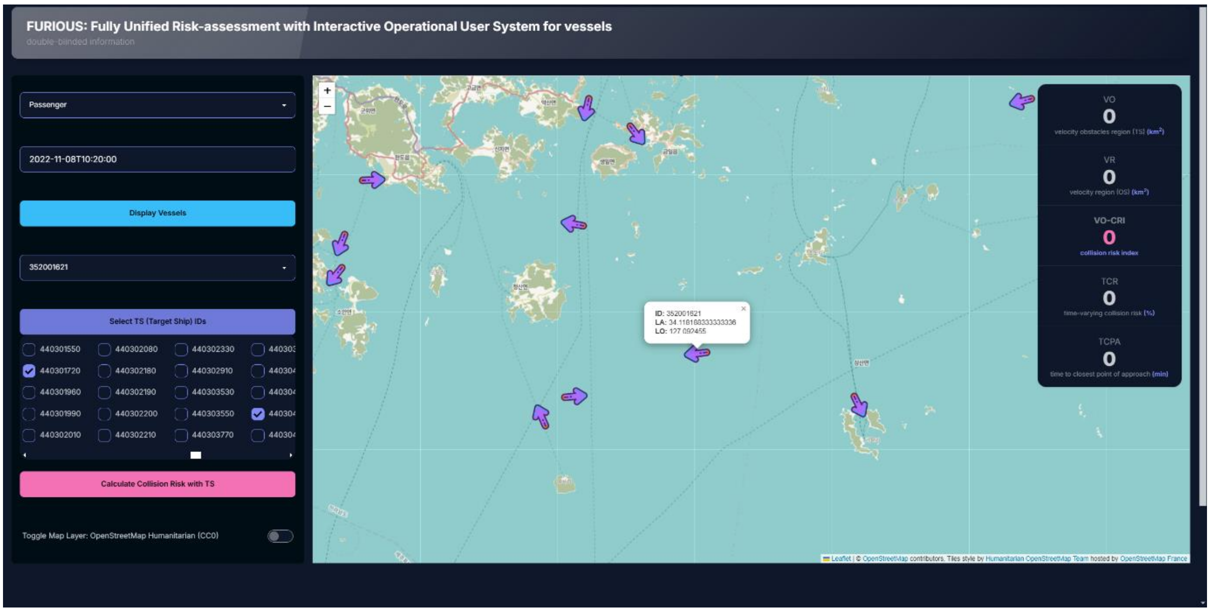

Supporting Information: Appendix

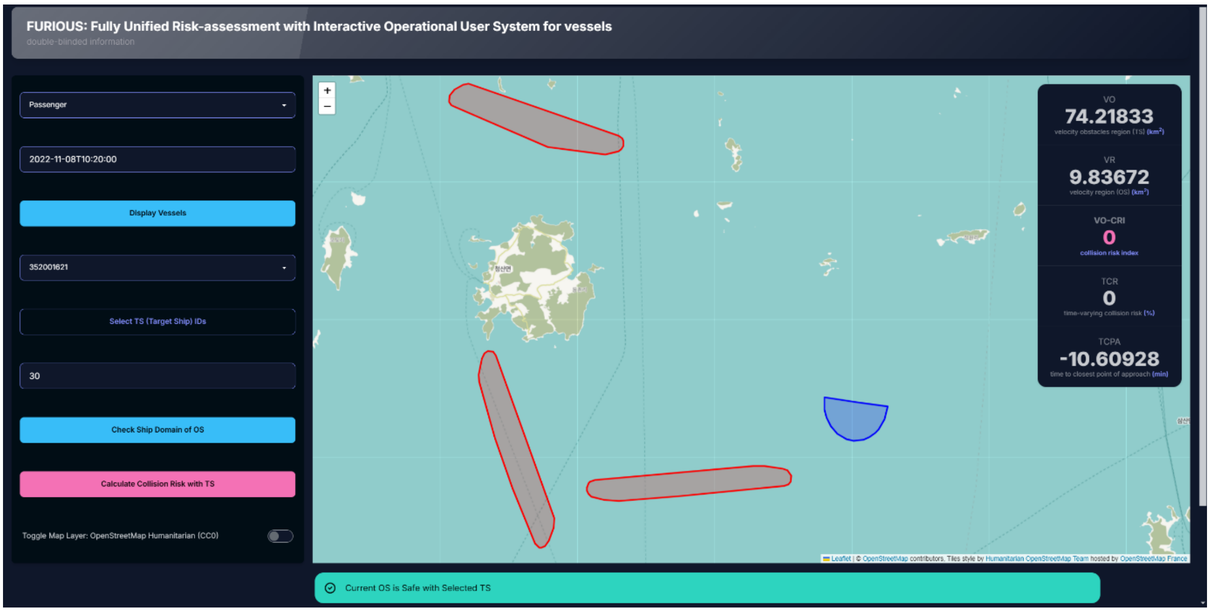

Ship Type	OS ID	TS IDs	Observation
passenger	440304410	440323420, 440335170, 440336350	2022-08-12 16:20 ~ 16:50

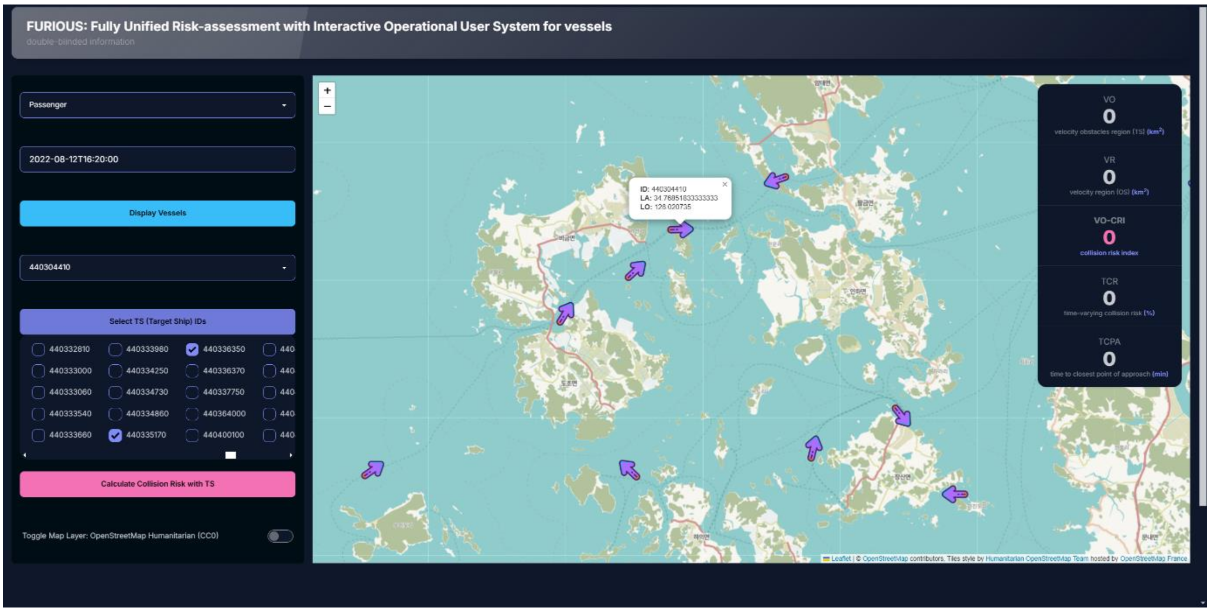

Supporting Information: Appendix

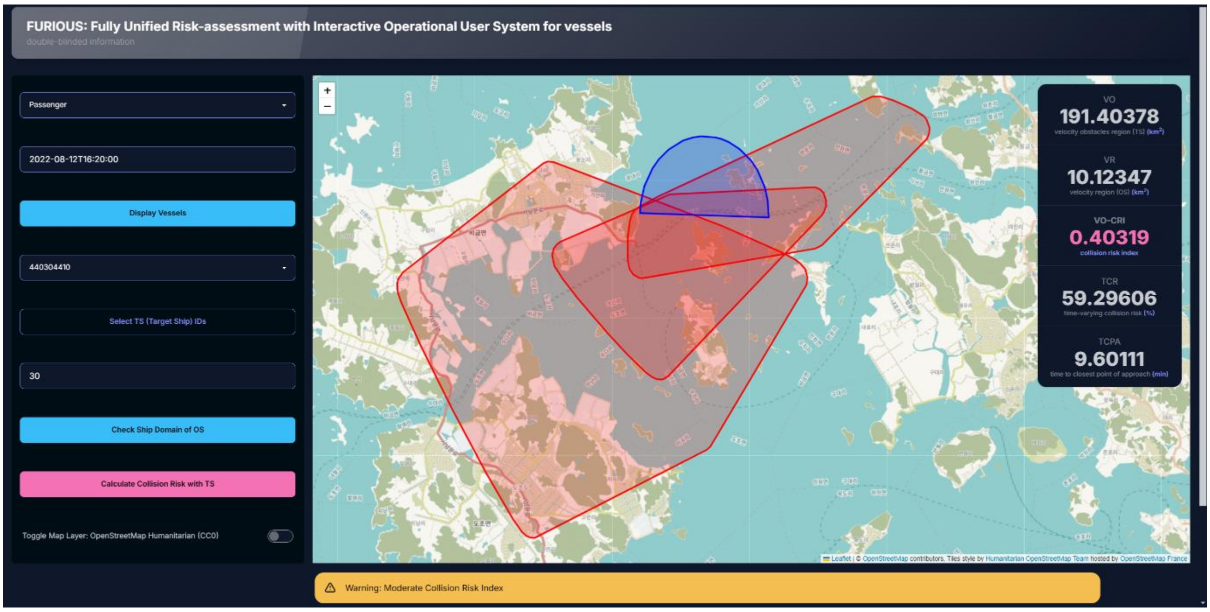

Ship Type	OS ID	TS IDs	Observation
cargo	538007317	636017001, 636015927, 671645000	2022-07-19 08:30 ~ 09:10

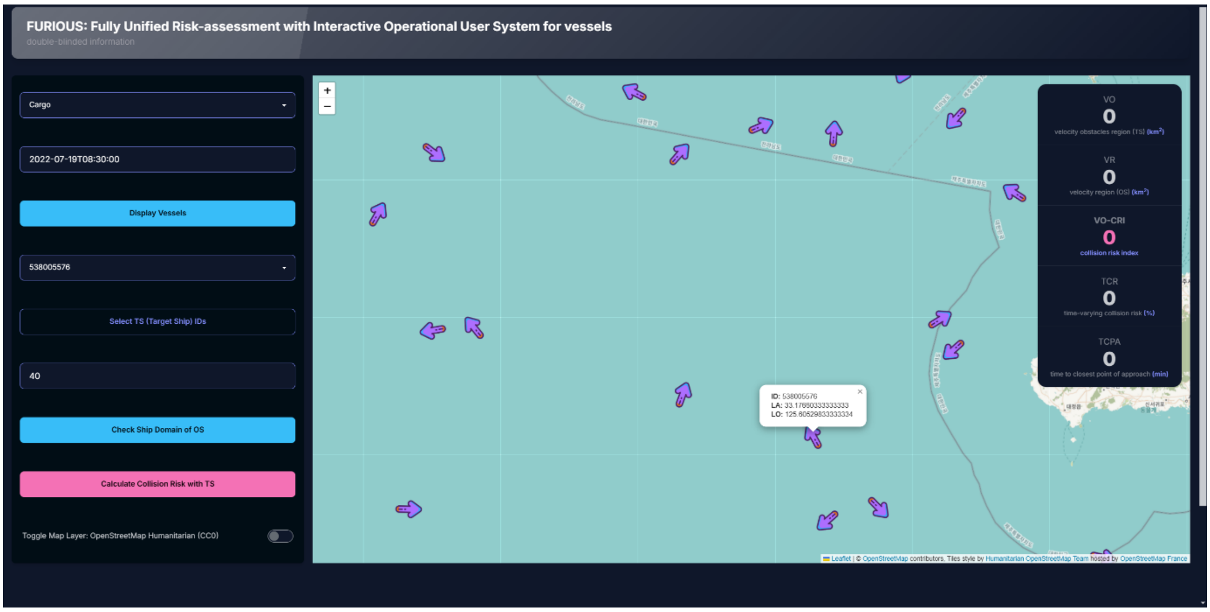

Supporting Information: Appendix

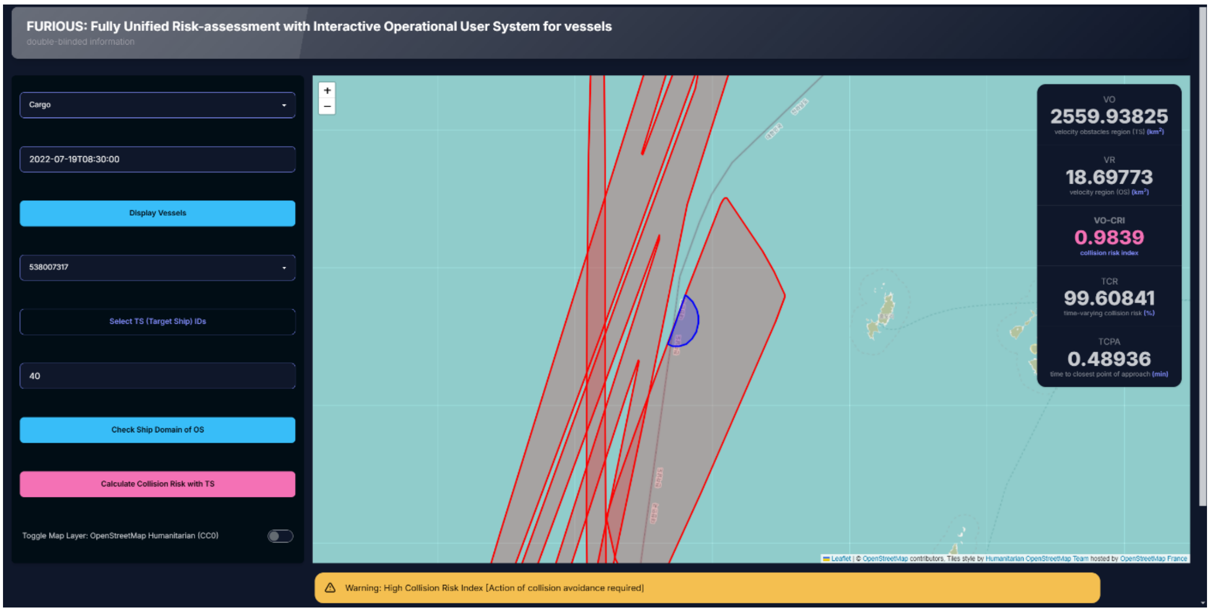

Supporting Information: Appendix

Screenshots of Dense Maritime Traffic Scenario

Ship Type	OS ID	TS IDs	Observation
passenger	440017010	440001420, 440001700, 440003940, 440009130, 440017940	2022-08-14 13:50 ~ 14:20

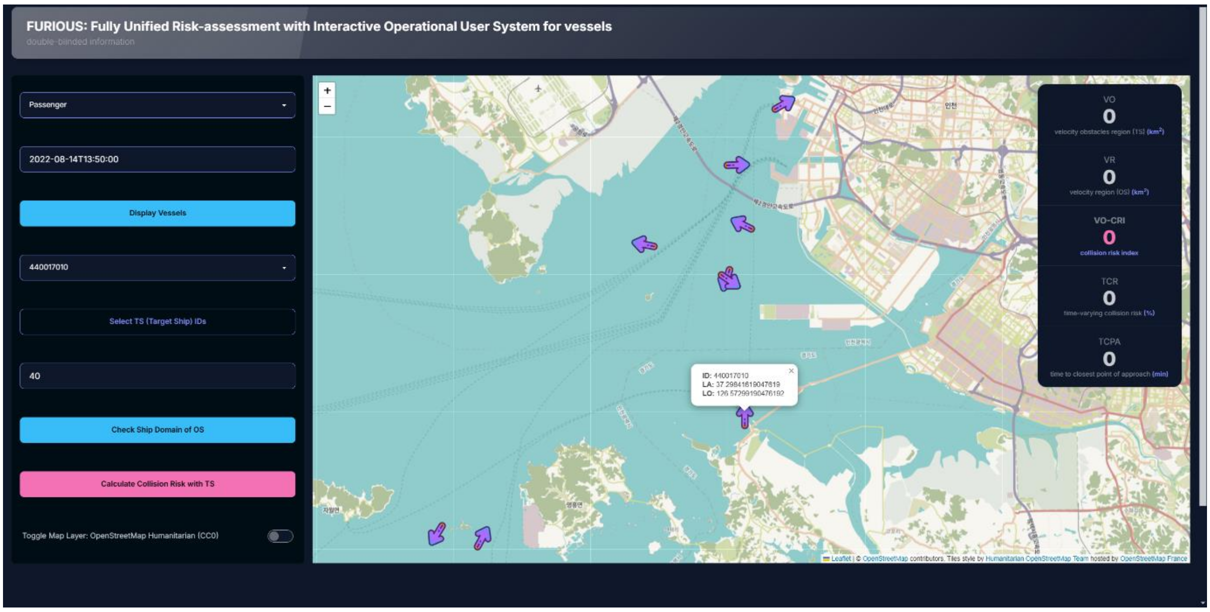

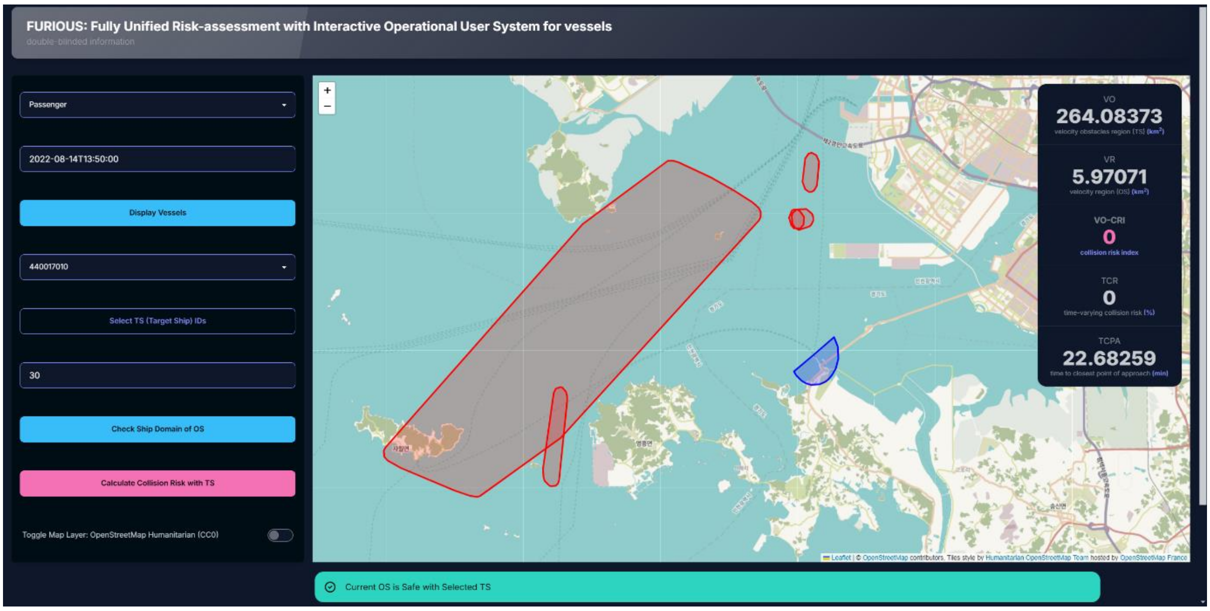

Supporting Information: Appendix

Ship Type	OS ID	TS IDs	Observation
passenger	440110060	440235000, 440294000, 440135170, 312291000, 440102620	2022-08-18 16:20 ~ 16:50

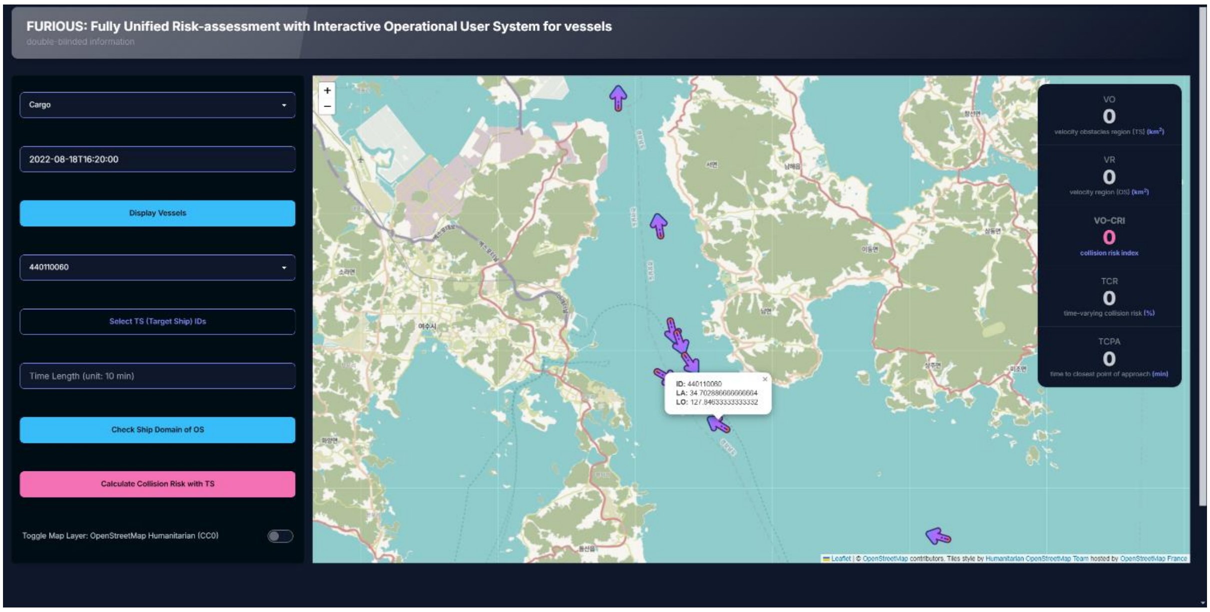

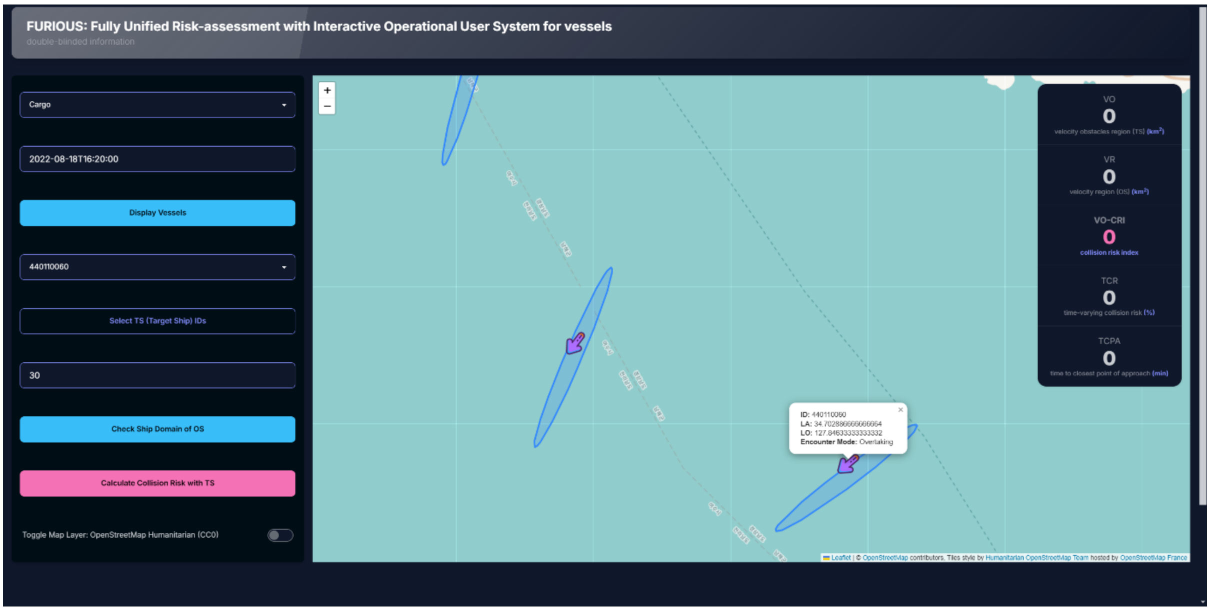

Supporting Information: Appendix

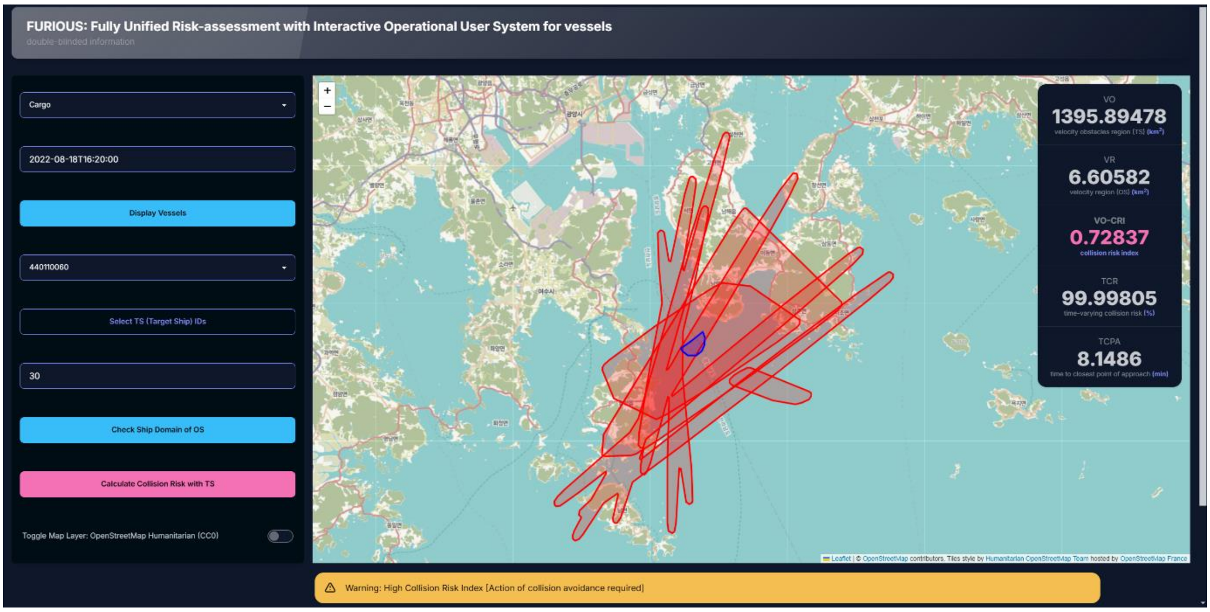

Supplement: S1 Appendix — (PDF) [file pone.0323300.s001.pdf]
