# Supplementary material for: FURIOUS: Fully unified risk-assessment with interactive operational user system for vessels
Source: PLoS One. 2025 May 28;20(5):e0323300. doi: 10.1371/journal.pone.0323300 (PMC12118981; doi:10.1371/journal.pone.0323300)
Supplement: S2 Appendix — This appendix contains the translated questions from the user survey conducted as part of the system evaluation of the “FURIOUS". (PDF) [file pone.0323300.s002.pdf]

User Survey (translated from Korean)

---

1. Background Information

1. What is your current major?
   - Data Science
   - Computer Engineering
   - Earth and Environmental Sciences
   - Naval Architecture and Ocean Engineering
   - Other: ______________________________
2. How many years of academic experience do you have in the fields of oceanography, geography, or related disciplines?
   - Less than 1 year
   - 1 to 2 years
   - 2 to 3 years
   - 3 to 4 years
   - More than 4 years
3. How many years of experience do you have in front-end development, such as visualization interfaces?
   - Less than 1 year
   - 1 to 2 years
   - 2 to 3 years
   - 3 to 4 years
   - More than 4 years
4. Have you had any experience using systems related to maritime route prediction?
   - Yes
   - No

---

2. System Usage Experience

1. How easy was it to use the system?
   - Not easy at all 1 2 3 4 5 Very easy
2. If you encountered any difficulties while using the system, please describe the main issues you faced.
3. How would you rate the response speed of the system?
   - Very slow 1 2 3 4 5 Very fast
4. Were the instructions provided for using the system sufficiently clear?
   - Not clear at all 1 2 3 4 5 Very clear

---

3. Interface Satisfaction

1. How intuitive did you find the interface?
    - Not intuitive at all 1 2 3 4 5 Very intuitive
2. How clear were the visualization results for the routes?
    - Not clear at all 1 2 3 4 5 Very clear
3. Were you satisfied with the interface design?
    - Not satisfied at all 1 2 3 4 5 Very satisfied

---

4. Visualization Comprehension

1. How useful were the visualization results for assessing collision risk?
    - Not useful at all 1 2 3 4 5 Very useful
2. Briefly describe which aspects of the visualization were useful (e.g., understanding of evaluation metrics, ship route analysis, etc.).
3. Could you trust the information presented in the visualizations?
    - Not trustworthy at all 1 2 3 4 5 Very trustworthy
4. Do you think the visualized information accurately reflected the real marine environment?
    - Not accurate at all 1 2 3 4 5 Very accurate

---

5. Overall Satisfaction

1. How satisfied were you with the system overall?
    - Not satisfied at all 1 2 3 4 5 Very satisfied
2. Would you use this system again in the future?
    - Not likely at all 1 2 3 4 5 Very likely
3. Would you recommend this system to others?
    - Not at all 1 2 3 4 5 Definitely

---

6. System Improvement and Additional Feedback

1. Are there any additional features you would like to see added to improve the system?
2. What do you think could be done to improve the user experience with the system?
3. Do you have any additional feedback you would like to provide?
