# Supplementary figures and images for: FURIOUS: Fully unified risk-assessment with interactive operational user system for vessels
Source: PLoS One. 2025 May 28;20(5):e0323300. doi: 10.1371/journal.pone.0323300 (PMC12118981; doi:10.1371/journal.pone.0323300)

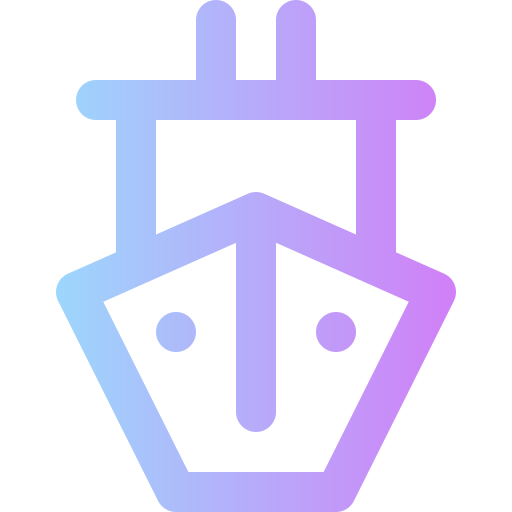

Supplement: S3 Appendix — This appendix provides a compressed ZIP archive containing the code developed by the authors. The same code is also accessible from the GitHub repository specified in the Code Availability section. (ZIP) [file pone.0323300.s003.zip › FURIOUS_PLOSONE-main/client/app/icon.png]

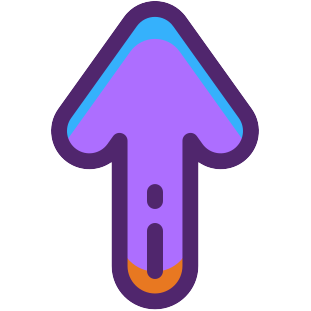

Supplement: S3 Appendix — This appendix provides a compressed ZIP archive containing the code developed by the authors. The same code is also accessible from the GitHub repository specified in the Code Availability section. (ZIP) [file pone.0323300.s003.zip › FURIOUS_PLOSONE-main/client/public/vesselarrow.png]
